# Small-Molecule Boron-10-Enriched Carriers with Exceptional Aqueous Solubility for Enhanced Boron Neutron Capture Therapy of Malignant Tumors

**DOI:** 10.34133/research.1315

**Published:** 2026-07-03

**Authors:** Tongyin Xiong, Xiangdi Yang, Tingting Li, Rongtao Song, Linxuan Huang, Xueli Sang, Fang Hu, Xiao Xu, Zhigang Liu

**Affiliations:** ^1^ Cancer Center, Guangdong Engineering Research Center of Boron Neutron Therapy and Application in Malignant Tumors, Dongguan Key Laboratory of Precision Diagnosis and Treatment for Tumors, The Tenth Affiliated Hospital, Southern Medical University (Dongguan People’s Hospital), Dongguan 523059, China.; ^2^ Biomaterials Research Center, School of Biomedical Engineering, Guangdong Provincial Key Laboratory of Medial Image Processing, Southern Medical University, Guangzhou 510515, China.

## Abstract

Boron neutron capture therapy (BNCT) enables localized tumor ablation while minimizing damage to surrounding tissues, offering advantages for treating anatomically challenging sites. However, current boron carriers, such as sodium borocaptate (^10^BSH), suffer from inadequate tumor specificity. Herein, the present study details the design, synthesis, and preclinical evaluation of a novel small-molecule boron-10-enriched carrier, which was synthesized by covalent bond coupling 4-carboxy-3-fluorophenylboronic acid (FPBA) to ^10^BSH (FPBA–BSH), achieving a boron content of approximately 25 wt.%. FPBA–BSH efficiently penetrated the blood–brain barrier and demonstrated pronounced accumulation in orthotopic gliomas, achieving a boron concentration of 75.4 μg-B/g-tumor tissue, which was 4.2- and 3.7-fold higher than those with boronophenylalanine (BPA) and BSH, respectively. Moreover, FPBA–BSH exhibited markedly improved tumor selectivity, with tumor-to-normal tissue (T/N) and tumor-to-blood ratios of 52.0 and 7.2, respectively. The T/N ratio was approximately 19.3- and 14.1-fold greater than those observed for BPA and BSH. In the melanoma model, FPBA–BSH achieved an intratumor boron concentration of 114.4 μg-B/g-tumor tissue, representing 8.4- and 9.9-fold increases compared with BPA and BSH, respectively. Correspondingly, the T/N and tumor-to-blood ratios reached 135.1 and 8.6, indicating substantially enhanced tumor targeting and retention. The T/N ratio achieved with FPBA–BSH was approximately 26.0- and 34.6-fold higher than those obtained with BPA and BSH, respectively. Consistent with its superior tumor selectivity, FPBA–BSH-mediated BNCT induced pronounced tumor-selective cytotoxicity and markedly inhibited tumor growth in both orthotopic glioma and melanoma models compared with BPA, BSH, and untreated controls. These findings demonstrate that FPBA–BSH represents a promising small-molecule boron delivery agent with substantial potential for clinical BNCT applications.

## Introduction

Boron neutron capture therapy (BNCT) is an emerging radiotherapy modality that facilitates precise, localized tumor ablation while sparing surrounding healthy tissues, making it an ideal strategy for managing tumors in anatomically complex regions [1,2]. Its therapeutic principle relies on the nuclear reaction between boron-10 and incident neutrons, which produces high linear energy transfer particles with ranges comparable to single-cell dimensions, thereby confining cytotoxicity to boron-containing cells [3–5]. The therapeutic effectiveness of BNCT relies on boron delivery agents capable of selectively accumulating within tumors while maintaining favorable tumor-to-normal tissue distribution [6–8]. Notably, the integration of BNCT with other therapeutic strategies has recently gained increasing attention [9,10].

Since the 1990s, the safety and therapeutic performance of BNCT have been investigated across multiple clinical centers in patients with glioblastoma, melanoma, and head-and-neck malignancies [11,12]. A landmark series of studies in the United States employed boronophenylalanine (BPA) as the boron carrier in combination with epithermal neutron beams, which provide greater tissue penetration and improved patient tolerance compared with thermal neutrons [13]. These early trials demonstrated that a single BNCT treatment could achieve survival outcomes comparable to fractionated radiotherapy while importantly reducing treatment time [14]. Such findings established the safety profile of BNCT and established the clinical feasibility of BPA-based treatment strategies employing high-flux neutron sources [15].

Despite these advances, currently available boron carriers remain suboptimal. Early boron-containing agents, including borax and boric acid, lacked tumor selectivity and produced systemic toxicity, whereas second-generation agents, primarily BPA and sodium borocaptate (BSH) [16], improved tumor uptake but are limited by low boron content, modest tumor specificity, poor blood–brain barrier (BBB) permeability, and short intratumoral retention [17–19]. BPA in its clinical formulation with fructose improves solubility but requires high dosing and carries risks of metabolic side effects [20,21]. To overcome these challenges, recent research has concentrated on the development of third-generation boron carrier systems, including small molecules with improved physicochemical properties, macromolecule-based carriers with enhanced targeting, and nanomedicine platforms designed for high boron payload and prolonged circulation [22–24]. Among these approaches, low-molecular-weight compounds that combine good aqueous solubility, high boron content, tumor selectivity, BBB penetration, and favorable pharmacokinetics are considered particularly attractive for clinical translation [25–27].

**Fig. 1.**
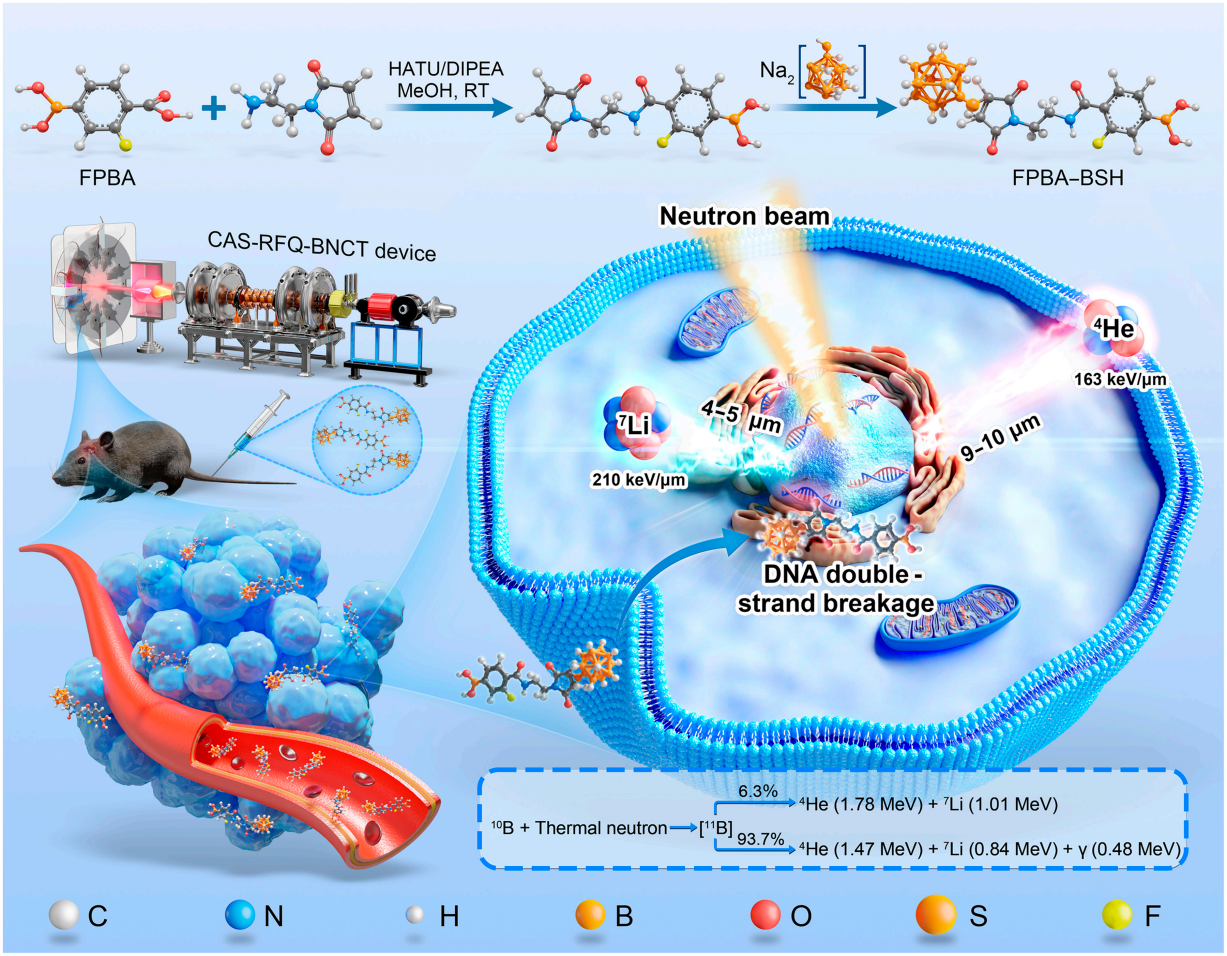
Schematic diagram of the synthesis of 4-carboxy-3-fluorophenylboronic acid (FPBA)–sodium borocaptate (BSH) and the mechanism of boron neutron capture therapy (BNCT) treatment. HATU, *N,N,N′,N′*-tetramethyl-*O*-(7-azabenzotriazol-1-yl)urea hexafluorophosphate; DIPEA, *N,N-*diisopropylethylamine. RT, room temperature; CAS, Chinese Academy of Sciences; RFQ, radio frequency quadrupole.

In this work, we synthesized a small-molecule boron-10-enriched carrier, 4-carboxy-3-fluorophenylboronic acid (FPBA)–BSH, by conjugating FPBA with BSH. This compound contains approximately 25 wt.% boron, which is 5 times higher than that of BPA. Following tail vein administration of the FPBA–BSH targeted delivery system in mice, ^10^B specifically accumulates within tumor tissue. Subsequent neutron beam irradiation induces nuclear fission reactions in locally absorbed ^10^B nuclei within the tumor, producing alpha particles (^4^He) with a high linear energy transfer of approximately 9 to 10 μm and lithium ions (^7^Li) with a linear energy transfer of approximately 4 to 5 μm. These short-range, high-energy particles directly interact with tumor cell DNA, causing double-strand breaks that lead to tumor cell apoptosis (Fig. 1). Mechanistic analyses indicate that phenylboronic acid groups mediate selective binding to sialic-acid-rich glycoconjugates on tumor cells [28,29]. FPBA–BSH-mediated BNCT produces tumor-selective cytotoxicity in glioma and melanoma models with minimal systemic toxicity. These findings demonstrate that FPBA–BSH combines high boron loading, favorable biodistribution, and selective tumor retention, representing a promising candidate for clinical translation as a next-generation boron carrier in BNCT.

## Results and Discussion

### Preparation of small-molecule boron drug FPBA–BSH

FPBA–BSH, a small molecular carrier enriched in ^10^B, was synthesized via a multistep organic reaction pathway (Fig. S1). The chemical structure and successful synthesis were confirmed by ^1^H nuclear magnetic resonance (^1^H NMR) spectroscopy and mass spectrometry (Figs. S2 and S3). Analytical high-performance liquid chromatography revealed a purity of 100%, indicating the compound’s suitability for subsequent biological and physicochemical evaluations (Fig. S4). FPBA–BSH yields a compound with approximately 25 wt.% boron—about 5 times higher than that of BPA. We have compiled recent literature-reported boron proportions in boron-containing agents (Table S1 and Fig. S5). As shown in Fig. S6, the log *P* value of FPBA–BSH falls within the range of 1 to 3, indicating that the compound possesses favorable cell-membrane permeability.

### Research on the intake mechanism

**Fig. 2.**
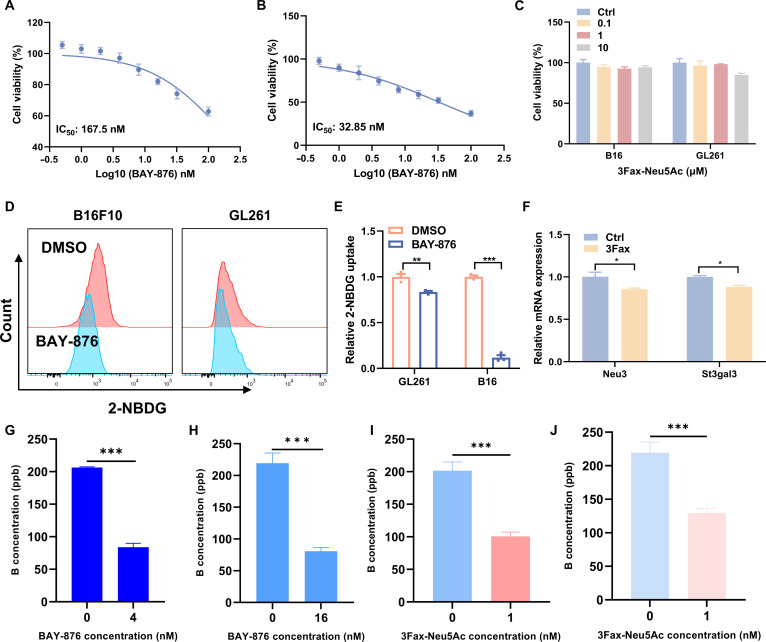
In vitro effects of GLUT1 inhibitor BAY-876 and sialic acid inhibitor 3Fax-Neu5Ac on B16F10 and GL261 cells. (A) The inhibitory effect of BAY-876 on B16F10 cell proliferation. (B) The inhibitory effect of BAY-876 on GL261 cell proliferation. (C) The effect of 3Fax-Neu5Ac on cell viability. (D) Histogram of 2-NBDG uptake detected by flow cytometry. DMSO, dimethyl sulfoxide. (E) Quantitative analysis of 2-NBDG intake. (F) Inhibition of sialic-acid-related gene expression by 3Fax-Neu5Ac. (G) The uptake of B16F10 cells treated with different concentrations of BAY-876 inhibitor. (H) The uptake of GL261 cells treated with different concentrations of BAY-876 inhibitor. (I) The uptake of B16F10 cells treated with different concentrations of 3Fax-Neu5Ac inhibitor. (J) The uptake of GL261cells treated with different concentrations of 3Fax-Neu5Ac inhibitor. *P < 0.05, **P < 0.01, ***P < 0.001 (statistical significance based on unpaired 2-tailed *t* test).

As shown in Fig. 2A and B, both B16-F10 murine melanoma cell line (B16F10) and GL261 murine glioblastoma cell line (GL261) cells were exposed to a range of BAY-876 (a selective GLUT1 inhibitor) concentrations to generate dose–response curves. GL261 cells exhibited markedly greater sensitivity to glucose transporter type 1 (GLUT1) inhibition than B16F10 cells, with a half maximal inhibitory concentration (IC_50_) of 32.85 nM, whereas B16F10 cells showed only minimal growth inhibition within the tested concentration range. Treatment with 3-fluoro-ax-*N*-acetylneuraminic acid (3Fax-Neu5Ac) (0.1 to 10 μM) did not induce detectable cytotoxicity in either cell line (Fig. 2C), consistent with its role as a metabolic inhibitor rather than a direct cytotoxin. BAY-876 significantly reduced glucose uptake in both cell lines (Fig. 2D and E), confirming effective GLUT1 blockade. In parallel, quantitative polymerase chain reaction (qPCR) analysis revealed that 3Fax-Neu5Ac treatment markedly decreased the mRNA levels of neuraminidase 3 (Neu3) and ST3 beta-galactoside alpha-2,3-sialyltransferase 3 (St3gal3) (Fig. 2F), indicating suppressed cellular sialylation. To further investigate whether GLUT1 activity and sialic acid biosynthesis contribute to FPBA–BSH internalization, intracellular boron concentrations were quantified following inhibitor pretreatment. As shown in Fig. 2G to J, treatment with BAY-876 (4 or 16 nM) markedly reduced intracellular boron content, decreasing from 219.2 ± 2.3 and 206.3 ± 5.2 ng/10^6^ cells to 80.8 ± 4.5 and 83.8 ± 2.4 ng/10^6^ cells, respectively. Likewise, 1 μM 3Fax-Neu5Ac similarly diminished FPBA–BSH accumulation, reducing it from 201.6 ± 3.2 and 219.1 ± 3.2 ng/10^6^ cells to 100.8 ± 2.5 and 129.5 ± 2.4 ng/10^6^ cells, respectively. To further investigate the effect of glucose on the uptake of FPBA–BSH, the cells were cultured in media containing high or low concentrations of glucose, and the amount of drug accumulation within the cells was quantitatively measured. As shown in Fig. S7, in the uptake experiments of GL261 cells with glucose concentration ranging from 5.5 to 25 mM (from low-sugar medium to high-sugar medium) and B16F10 cells with glucose concentration ranging from 5.5 to 11 mM (from low-sugar medium to high-sugar medium), we observed that as the extracellular glucose concentration in B16F10 and GL261 cells increased, the accumulation of FPBA–BSH in B16F10 cells significantly increased to 110.3 ppb, which was 1.2 times that of low glucose; the accumulation in GL261 cells significantly increased to 199.2 ppb, which was twice that of low glucose, indicating that its uptake level significantly decreased under low-glucose conditions. This result indicates that the extracellular glucose level affects the internalization efficiency of FPBA–BSH, supporting the role of glucose-transport-related pathways in its intracellular uptake. Collectively, these results indicate that both GLUT1-mediated transport and cellular sialylation influence the uptake of FPBA–BSH. To further validate the involvement of GLUT1 in cellular uptake, Slc2a1 was silenced using 3 independent siRNAs in B16F10 and GL261 cells. qPCR analysis confirmed effective knockdown, with siGLUT1-3 showing the highest silencing efficiency in both cell lines. In B16F10 cells, GLUT1 mRNA levels were reduced by 29.9%, 88.3%, and 73.9% in the siGLUT1-1 (small interfering RNA targeting GLUT1, sequence 1), siGLUT1-3 (small interfering RNA targeting GLUT1, sequence 3), and siGLUT1-5 (small interfering RNA targeting GLUT1, sequence 5) groups, respectively (Fig. S8A). In GL261 cells, corresponding reductions of 11.5%, 44.3%, and 31.6% were observed (Fig. S8B). Consistent with the knockdown efficiency, GLUT1 silencing markedly reduced intracellular boron accumulation. In B16F10 cells, boron concentration decreased from 258.6 ppb in the small interfering RNA negative control group to 165.9, 132.1, and 161.5 ppb following transfection with siGLUT1-1, siGLUT1-3, and siGLUT1-5, respectively (Fig. S8C). A similar trend was observed in GL261 cells, where intracellular boron levels decreased from 311.8 ppb in the control group to 186.0, 168.0, and 188.1 ppb, respectively (Fig. S8D). Notably, siGLUT1-3, which produced the greatest reduction in GLUT1 expression, also resulted in the most pronounced decrease in boron uptake in both cell lines. These results indicate that there is a positive correlation between the expression of GLUT1 and the uptake of FPBA–BSH, thereby suggesting that GLUT1 plays a major role in the cellular transport of FPBA–BSH. While these findings support a functional contribution of glucose transport and surface glycosylation to FPBA–BSH internalization, further mechanistic studies will be required to delineate the specific molecular pathways involved.

### Cytotoxicity and cellular uptake of FPBA–BSH

**Fig. 3.**
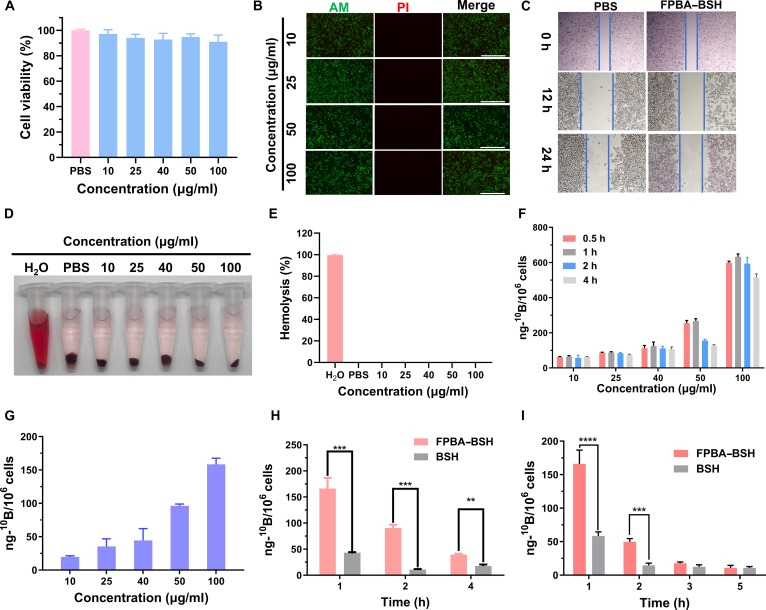
Cytotoxicity and cellular uptake properties of FPBA–BSH. (A) Relative viability of B16F10 cells after 48-h incubation with varying concentrations of FPBA–BSH, assessed by Cell Counting Kit-8 (CCK-8) assay. PBS, phosphate-buffered saline. (B) Representative fluorescence images of live/dead-stained B16F10 cells following treatment with FPBA–BSH (Calcein-acetoxymethyl ester [Calcein-AM]/propidium iodide [PI]). Scale bar = 200 μm. (C) Quantification of cell migration capacity after FPBA–BSH exposure based on scratch assay. (D and E) Hemolysis analysis of FPBA–BSH at different boron concentrations (0 to 100 μg/ml); data represent absorbance-based quantification relative to negative and positive controls. (F) Time- and dose-dependent intracellular boron accumulation in GL261 cells after incubation with FPBA–BSH for 1, 2, and 4 h, as measured by inductively coupled plasma mass spectrometry (mean ± SD, *n* = 3). (G) Intracellular boron levels in B16F10 cells following 1-h incubation with FPBA–BSH at indicated boron concentrations (mean ± SD, *n* = 3). (H) Comparative cellular uptake of FPBA–BSH and BSH in B16F10 cells at an equivalent boron dose after incubation for 1 h. (I) Boron retention profiles in B16F10 cells post-incubation with FPBA–BSH or BSH at 100 μg/ml for 1 h, followed by washing and further incubation in fresh medium for indicated time points. **P < 0.01, ***P < 0.001, ****P < 0.0001 (statistical significance based on unpaired 2-tailed *t* test).

FPBA–BSH demonstrates favorable biocompatibility across a range of in vitro assays. To assess its intrinsic cytotoxicity, GL261 and B16F10 cells were exposed to different concentrations of FPBA–BSH (Fig. 3A and Fig. S9) for 48 h, followed by Cell Counting Kit-8 (CCK-8) viability assays. Cell viability remained above 90% at all tested concentrations, including up to 100 μg/ml of FPBA–BSH, indicating negligible cytotoxic effects. These findings were corroborated by Calcein-acetoxymethyl ester (Calcein-AM)/propidium iodide (PI) staining, which similarly showed no signs of cytotoxicity (Fig. 3B). Even in the presence of serum, B16F10 cells failed to close wounds in the scratch assay regardless of FPBA–BSH addition, indicating that FPBA–BSH did not significantly affect cell migration capacity (Fig. 3C).

To evaluate the hemocompatibility of FPBA–BSH, a hemolysis assay was conducted. While the positive control induced significant erythrocyte lysis, no appreciable hemolysis was observed following 3-h incubation with FPBA–BSH at concentrations between 0 and 100 μg/ml, as compared to the saline negative control (Fig. 3D). Quantitative hemolysis analysis, based on supernatant absorbance at 540 nm, revealed hemolysis rates consistently below 1% across all tested concentrations, confirming the excellent hemocompatibility of FPBA–BSH as a boron carrier (Fig. 3E). These results collectively confirm that FPBA–BSH is minimally toxic and exhibits high biocompatibility, making it suitable for biomedical applications such as BNCT. To investigate cellular uptake efficiency, GL261 cells were incubated with FPBA–BSH at varying concentrations (10, 25, 40, 50, and 100 μg/ml) for 1, 2, and 4 h. After washing with phosphate-buffered saline (PBS), intracellular boron content was quantified via inductively coupled plasma mass spectrometry (ICP-MS). The maximal boron accumulation (632.9 ± 3.1 ng/10^6^ cells) was observed at 1 h, indicating rapid cellular uptake and a dose-dependent increase in boron concentration (Fig. 3F). Similar uptake kinetics were observed in B16F10 cells, where the highest boron concentration reached 158.3 ± 10.5 ng/10^6^ cells at 1 h (Fig. 3G). In both cell lines, the boron concentration within the cells meets the clinical requirements for BNCT [6].

To directly assess comparative uptake, B16F10 cells were exposed to FPBA–BSH or BSH (100 μg/ml boron) for 1 to 4 h. ICP-MS analysis revealed substantially higher intracellular boron in FPBA–BSH-treated cells, with levels approaching 4-fold those obtained with BSH, highlighting the markedly superior uptake efficiency of FPBA–BSH (Fig. 3H). As shown in Fig. 3I, under a boron concentration of 100 μg/ml boron, FPBA–BSH achieved an intracellular boron concentration of 166.1 ± 10.5 ng/10^6^ cells within 1 h. This value is approximately 3 times that observed when using BSH. Importantly, FPBA–BSH maintained a boron concentration of 17.7 ± 2.1 ng/10^6^ cells at 3 h, still meeting the clinical requirements of BNCT [6], while the boron content in the BSH group had already fallen below this requirement after 2 h. These results highlight the improved cellular uptake and retention of FPBA–BSH relative to BSH, further supporting its promise as an advanced boron delivery agent for BNCT.

### Biochemical mechanism of FPBA–BSH performed on B16F10 cells

**Fig. 4.**
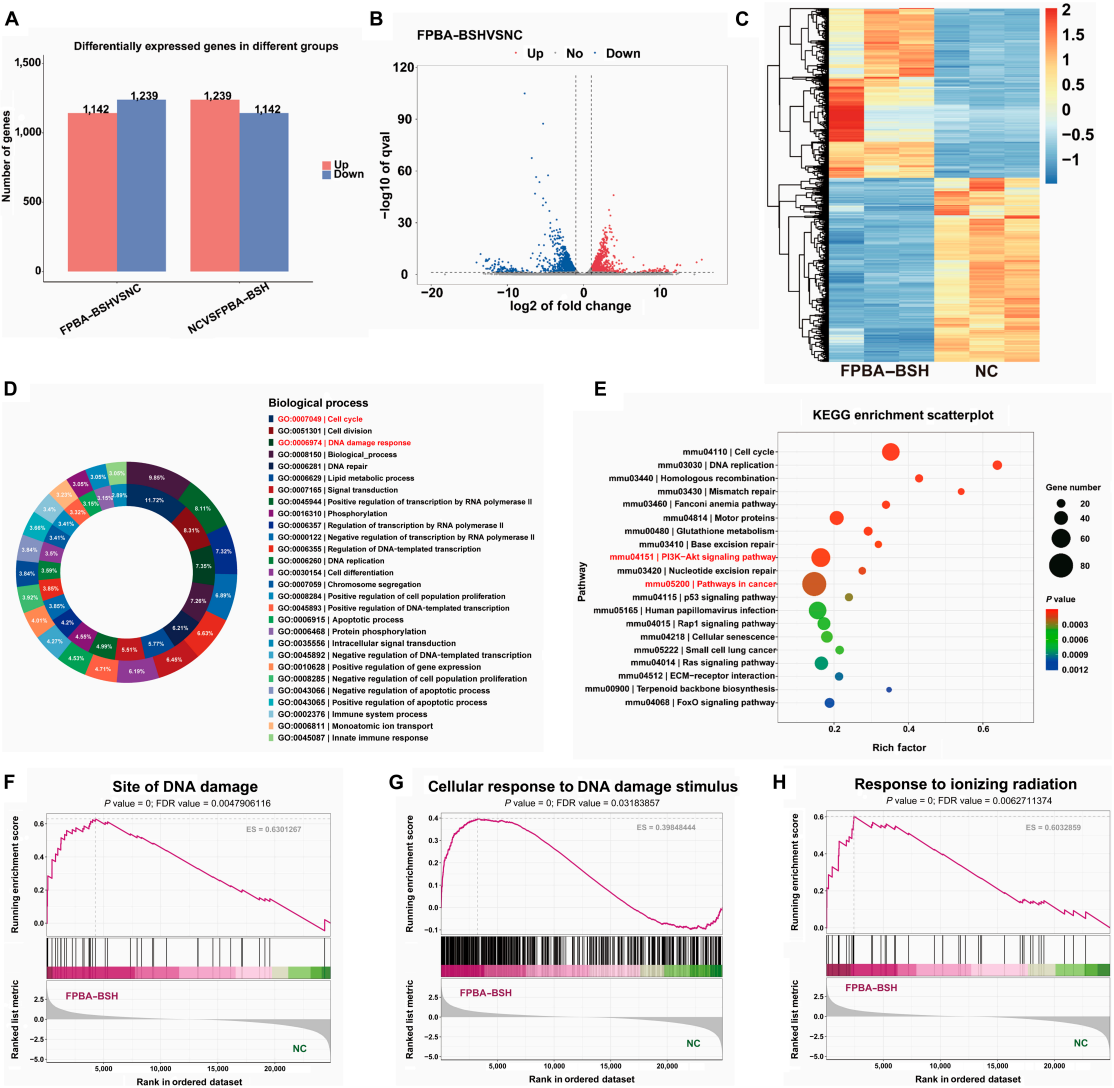
FPBA–BSH mRNA sequencing analysis. (A) Statistical graph of the number of differentially expressed genes. (B) Volcano plot of differentially expressed genes.(C) Heatmap of differentially expressed genes. (D) Gene Ontology (GO) functional enrichment analysis. (E) Scatter plot of Kyoto Encyclopedia of Genes and Genomes (KEGG) pathway enrichment analysis. (F to H) Gene set enrichment analysis results. FPBA–BSH, boron-delivery-agent-treated experimental group; NC, negative control group; FPBA–BSHVSNC, FPBA–BSH-treated group versus negative control group; ECM, extracellular matrix; FDR, false discovery rate; ES, enrichment score.

In order to conduct a more in-depth study on the killing effect of FPBA–BSH on B16F10 cells after neutron irradiation and to conduct sequencing analysis on the mRNA of B16F10 cells before and after irradiation, as shown in Fig. 4A, a total of 1,142 genes were up-regulated and 1,239 genes were down-regulated in the FPBA–BSH group compared with the NC group, indicating extensive differential gene expression. The volcano plot further illustrates the distribution of differentially expressed genes (Fig. 4B). Hierarchical clustering of the differentially expressed genes revealed distinct expression patterns between FPBA–BSH and NC samples (Fig. 4C). The heatmap shows clear segregation of the 2 groups, demonstrating consistent transcriptional differences between treatment and control samples. To clearly identify the functions controlled by these genes, Gene Ontology analysis was used to study the signaling pathways involved in the differentially expressed genes after neutron treatment in FPBA–BSH, and an enrichment network diagram was drawn. In Fig. 4D, the functions of the differentially expressed genes mainly concentrated in the cell cycle, DNA repair, and cancer pathways. Subsequently, the functions of these differentially expressed genes were further studied through the Kyoto Encyclopedia of Genes and Genomes enrichment analysis. The identified differentially expressed genes were mainly concentrated in the phosphoinositide 3-kinase–Akt signaling pathway, nucleotide excision repair, and cancer pathways (Fig. 4E). In Fig. 4F to H, the correlations between ionizing radiation responses, cellular responses to DNA damage stimuli, and genes at DNA damage sites are all markedly positive. Under neutron irradiation, FPBA–BSH can induce cell apoptosis and activate the corresponding apoptotic pathways, demonstrating a strong cytotoxic effect.

### Evaluation of BNCT efficacy and cellular uptake mechanism of FPBA–BSH

**Fig. 5.**
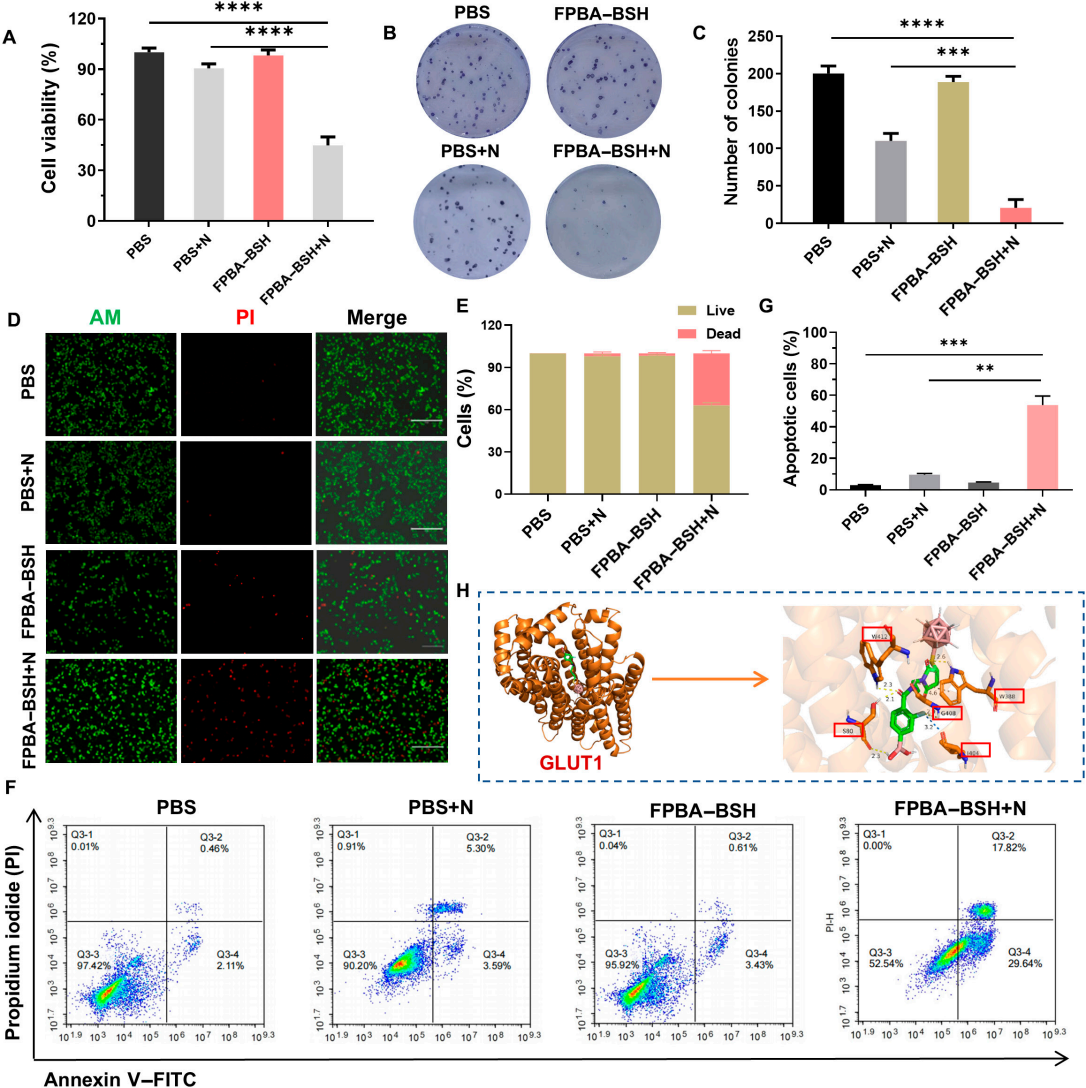
In vitro evaluation of BNCT efficacy and uptake mechanism of FPBA–BSH in B16F10 cells. (A) Cell viability of B16F10 melanoma cells following various treatments with or without neutron irradiation, as determined by the CCK-8 assay (mean ± SD, *n* = 3). (B) Representative images of colony formation assays under different treatment conditions. (C) Quantitative analysis of colony numbers corresponding to panel B. (D) Live/dead cell staining using Calcein-AM/propidium iodide (PI) after different treatments. Scale bar = 200 μm. (E) Quantification of viable and dead cells from panel D. (F) Flow cytometry analysis of apoptosis in B16F10 cells subjected to the indicated treatments. (G) Quantitative evaluation of apoptotic cell populations from panel F. (H) Molecular docking simulations illustrating the predicted binding interactions between FPBA–BSH and the GLUT1 transporter, with detailed views of hydrogen-bonding networks. Statistical significance: **P < 0.01, ***P < 0.001, ****P < 0.0001 (statistical significance based on unpaired 2-tailed *t* test).

As shown in Fig. 5A, pronounced cytotoxicity was evident exclusively in the FPBA–BSH + neutron group, whereas all other controls exhibited negligible effects. This trend was corroborated by colony formation assays (Fig. 5B), which demonstrated a striking reduction in clonogenic survival following BNCT (Fig. 5C). Consistently, live/dead staining (Fig. 5D) revealed a sharp decline in cell viability, with only ~63% of cells remaining viable after BNCT (Fig. 5E), while FPBA–BSH alone caused minimal damage. Flow cytometric analysis further substantiated these results, showing a robust apoptotic response (47.5%) in FPBA–BSH-treated cells exposed to neutrons (Fig. 5F and G). These results collectively demonstrate the robust antitumor potential of FPBA–BSH when combined with neutron irradiation. To elucidate the underlying mechanism governing the interaction between FPBA–BSH and its potential cellular targets, molecular docking simulations were performed. As illustrated in Fig. 5H and Fig. S10, FPBA–BSH exhibited a strong predicted binding affinity to the glucose transporter protein GLUT1, with a calculated binding free energy of −7.56 kcal/mol. This was substantially lower than its binding affinity to the macrophage scavenger receptor sialic acid-binding immunoglobulin-like lectin 1 (−4.33 kcal/mol), as summarized in Table S2. Detailed structural analysis of the docking complex revealed an extensive hydrogen-bonding network between FPBA–BSH and key residues within the GLUT1 binding pocket, supporting the hypothesis that GLUT1 may serve as a major mediator of FPBA–BSH cellular uptake [30]. Together, these findings suggest that FPBA–BSH can effectively mediate boron delivery to tumor cells and enable potent BNCT efficacy, likely facilitated by GLUT1-mediated transport mechanisms.

### Evaluation of BNCT efficacy in GL261 cells in vitro

**Fig. 6.**
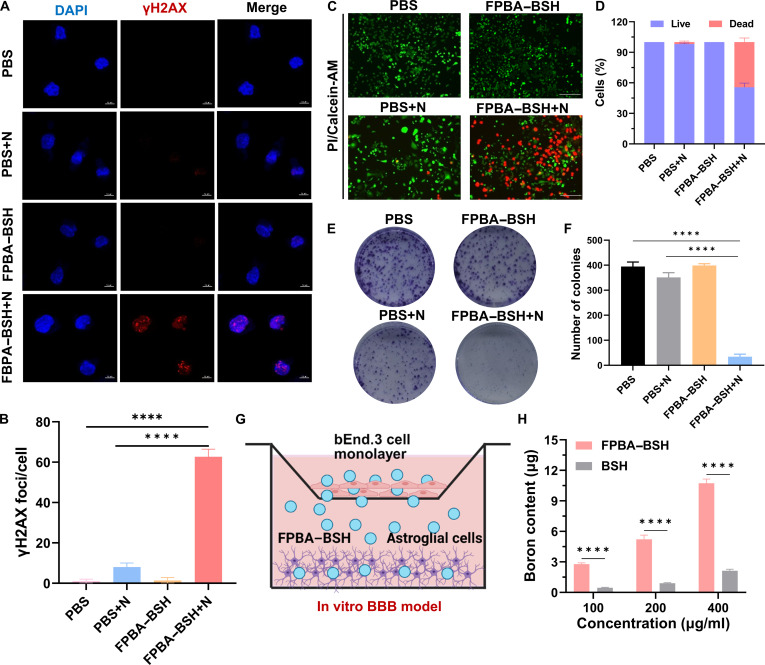
Evaluation of BNCT treatment in GL261 cells in vitro. (A) γH2AX immunofluorescence staining of GL261 cells following various treatment conditions under BNCT. Scale bar = 12 μm. (B) Quantification of γH2AX foci per nucleus across treatment groups. (C) Representative Calcein-AM/PI live–dead staining images of GL261 cells posttreatment. Scale bar = 200 μm. (D) Quantitative analysis of cell viability derived from Calcein-AM/PI staining in panel C. (E) Colony formation assay assessing clonogenic survival of GL261 cells after different treatments. (F) Statistical analysis of colony numbers from panel E. (G) In vitro model simulating blood–brain barrier (BBB) penetration. (H) Quantitative measurement of boron content in the lower chamber across different treatment concentrations. Statistical significance: ****P < 0.0001 (statistical significance based on unpaired 2-tailed *t* test), *n* >3.

To rigorously validate the efficacy of FPBA–BSH-enhanced BNCT in GL261 glioma cells in vitro, we employed gamma histone H2A variant X (γH2AX) immunostaining post-irradiation to quantify DNA double-strand breaks. As illustrated in Fig. 6A and B, cells treated with FPBA–BSH combined with neutron irradiation exhibited a markedly higher incidence of double-strand breaks compared to controls treated with PBS, PBS plus neutrons, or FPBA–BSH alone. Furthermore, through live/dead staining (Fig. 6C and D), it was shown that only in the FPBA–BSH+N group was there significant cytotoxicity, with cell viability reduced to approximately 55%, while the control group showed no toxicity. This effect was also confirmed by the colony formation experiment (Fig. 6E and F), which demonstrated a significant decrease in clone formation survival rate after combined treatment, highlighting the potent cytotoxicity of FPBA–BSH under neutron irradiation. To further evaluate its application potential, an in vitro BBB penetration model was established (Fig. 6G) [31]. Quantitative ICP-MS analysis of the lateral substrate region revealed markedly higher boron transport efficiency for FPBA–BSH compared with BSH (Fig. 6H), indicating superior BBB permeability. Collectively, these results demonstrate that FPBA–BSH not only substantially enhances BNCT efficacy through increased DNA damage and cytotoxicity but also exhibits markedly improved BBB penetration, highlighting its therapeutic potential against glioma.

### Therapeutic efficacy of FPBA–BSH-enhanced BNCT for melanoma

**Fig. 7.**
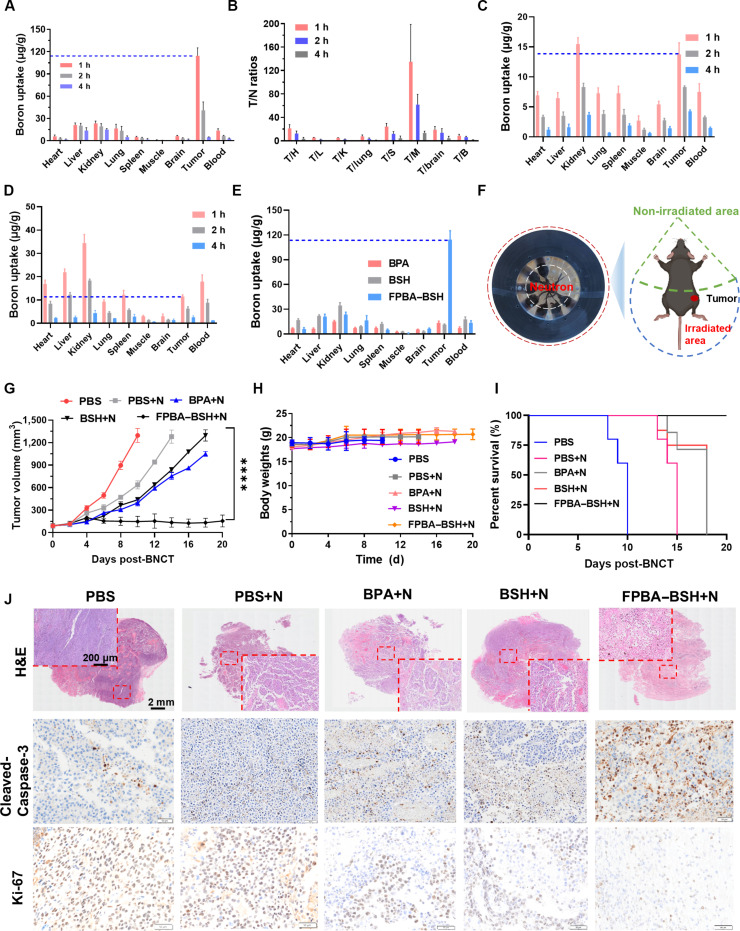
FPBA–BSH in BNCT for melanoma. (A) Boron concentrations in major organs and tumor tissues at 1, 2, and 4 h post-intravenous injection of FPBA–BSH (mean ± SD, *n* = 3). (B) T/N ratios calculated from data in panel A (mean ± SD, *n* = 3). (C) Boron concentrations in major organs and tumor tissues at 1, 2, and 4 h post-intravenous injection of BPA (mean ± SD, *n* = 3). (D) Boron concentrations in major organs and tumor tissues at 1, 2, and 4 h post-intravenous injection of BSH (mean ± SD, *n* = 3). (E) Boron concentrations in major organs and tumor tissues at 1 h post-intravenous injection of BPA, BSH, and FPBA–BSH (mean ± SD, *n* = 3). (F) Photograph of a mouse secured in a custom-designed mold for precise neutron irradiation; red dashed lines indicate tissues within the radiation field, while the area between blue and green dashed lines denotes the designated radiation zone. (G to I) Therapeutic evaluation in B16F10 melanoma-bearing female C57BL/6J mice treated with PBS, PBS+N, BPA+N, BSH+N, or FPBA–BSH+N (*n* = 6). Therapeutic intervention commenced once tumor volumes reached approximately 50 to 100 mm^3^. (G) Tumor growth curves showing mean tumor volumes over 20 d posttreatment. Statistical significance: ****P < 0.0001 (statistical significance based on unpaired 2-tailed *t* test), *n* = 6. (H) Mouse body weight monitored 20 d posttreatment (mean ± SD, *n* = 6). (I) Kaplan–Meier survival analysis of each treatment group. (J) Representative histological images of tumor sections stained with hematoxylin and eosin (H&E), Ki-67, and cleaved Caspase-3 after treatment. Scale bar = 50 μm.

To comprehensively assess the therapeutic potential of the FPBA–BSH boron compound in BNCT, we investigated its in vivo biodistribution following intravenous administration at a dose of 300 mg/kg via the tail vein. Notably, boron accumulation peaked at the tumor site, reaching an average concentration of 114.4 μg/g at 1 h postinjection (Fig. 7A). Comparative analysis of boron uptake in tumors over recent years revealed that FPBA–BSH achieves markedly higher intratumoral boron levels relative to previously reported agents, satisfying clinical threshold requirements (Fig. S11). The tumor-to-normal tissue (T/N) ratios exceeded 3 for lung, heart, brain, and muscle tissues (Fig. 7B), indicating a favorable therapeutic window with minimized off-target radiation exposure. Subsequently, a systematic assessment of the boron distribution of BPA and BSH in the body was conducted. One hour after intravenous administration, the boron concentration in the tumors of the BPA group reached 13.7 μg/g (Fig. 7C), and that of the BSH group reached 11.5 μg/g (Fig. 7D). In both cases, only the corresponding tumor-to-muscle (T/M) ratios exceeded 3.0 (Fig. S12A and B), indicating a certain tumor selectivity. Surprisingly, FPBA–BSH showed significantly higher tumor accumulation: at the same time point (1 h), its boron concentration in the tumors was 8.4 times that of BPA and 9.9 times that of BSH (Fig. 7E). Additionally, the T/N ratios of FPBA–BSH relative to both boron carriers were significantly increased (Fig. S12C). Among them, the T/M ratios for BPA, BSH, and FPBA–BSH were 5.2, 3.9, and 135.1, respectively. Notably, the T/M ratio of FPBA–BSH was approximately 26.0-fold and 34.6-fold higher than those of BPA and BSH, respectively, indicating a markedly enhanced tumor selectivity. Finally, the blood circulation behavior of FPBA–BSH was evaluated. As shown in Fig. S13 and Table S3, the blood pharmacokinetics of BPA, BSH, and FPBA–BSH were assessed by measuring boron concentrations at multiple time points after administration. All 3 agents reached their maximum blood levels at 1 h, with concentrations of 3.9, 18.3, and 21.0 μg/ml for BPA, BSH, and FPBA–BSH, respectively. Pharmacokinetic analysis showed that the blood half-lives of BPA, BSH, and FPBA–BSH were 58.2, 115.5, and 210.0 min, respectively. Notably, FPBA–BSH displayed a markedly prolonged circulation profile, with a half-life approximately 3.6-fold longer than that of BPA and 1.8-fold longer than that of BSH. In parallel, the systemic clearance of FPBA–BSH was significantly reduced, being ~6.6-fold lower than that of BPA and ~2.0-fold lower than that of BSH. These results indicate that FPBA–BSH possesses enhanced in vivo retention and improved pharmacokinetic behavior, which may facilitate increased tumor accumulation and thereby benefit BNCT efficacy.

During the irradiation process, the anesthetized mice were positioned in a lateral posture within a custom-fabricated circular arrangement device, with their thighs facing the center of the neutron beam. This setup ensured that the tumors could be exposed to the thermal neutrons, while the liver, spleen, and intestines were shielded from radiation (Fig. 7F). To evaluate antitumor efficacy in vivo, C57BL/6J female mice bearing melanoma xenografts received intravenous injections of either saline (100 μl), BPA, BSH, or FPBA–BSH (300 mg/kg) 1 h prior to neutron irradiation at a neutron flux of 0.8 × 10^9^ n·cm^−2^·s^−1^. Under 30-min neutron irradiation, the tumor boron concentration of FPBA–BSH reached 114.4 ppm, corresponding to a calculated boron dose of approximately 12.2 Gy. The resulting compound biological effectiveness (CBE) was estimated to be ~3.9, slightly higher than that of conventional BPA, indicating a modest improvement in biological effectiveness. Control cohorts included saline-injected mice with and without neutron exposure. Tumor growth, body weight, and survival were longitudinally monitored following treatment. In the saline group, tumor volume expanded from 91.2 ± 4.3 mm^3^ to beyond 1,200 mm^3^ within 10 d, while neutron irradiation alone delayed progression only modestly, reaching over 1,200 mm^3^ in 14 d; after being subjected to neutron irradiation, the tumor volume of BSH increased by more than 1,200 mm^3^ within 18 d, while the tumor volume in the BPA group treated with neutrons showed a slight inhibition (Fig. 7G). By contrast, the FPBA–BSH+N group almost completely suppressed tumor growth, limiting volumes to about 160 mm^3^ after 20 d, serum biochemistry (aspartate transaminase, alanine transaminase, creatinine, and urea) and ex vivo tumor weights were recorded at endpoint. Notably, body weight remained stable throughout treatment and the serum indicators did not show any significant changes, indicating minimal systemic toxicity (Fig. 7H and Fig. S14). Survival analysis revealed that all PBS and PBS+N groups succumbed within 10 d, BPA+N and BSH+N groups succumbed within 18 d, whereas mice receiving FPBA–BSH+N exhibited significantly extended survival beyond 20 d (Fig. 7I).

Histopathological and molecular analyses corroborated these findings. Immunohistochemical staining revealed elevated cleaved Caspase-3 expression in tumors from the FPBA–BSH+N group compared to PBS, PBS+N, BPA+N, and BSH+N, indicative of enhanced apoptosis and DNA damage (Fig. 7J). Concurrently, Ki-67 staining and hematoxylin and eosin (H&E) assays demonstrated a marked reduction in proliferative tumor cells and overall tumor cellularity following combined treatment (Fig. S15A and B), consistent with in vitro observations and highlighting the synergistic tumor-suppressive effects of FPBA–BSH-mediated BNCT. Crucially, H&E examination of major organs (heart, liver, spleen, lungs, and kidneys) revealed no histopathological abnormalities relative to controls (Fig. S16), underscoring the biosafety profile of FPBA–BSH. Collectively, these data validate the pronounced antitumor activity accompanied by a favorable safety profile of FPBA–BSH as a boron delivery agent in BNCT, highlighting its translational potential for melanoma therapy.

### Therapeutic evaluation of FPBA–BSH in BNCT for orthotopic glioma model

**Fig. 8.**
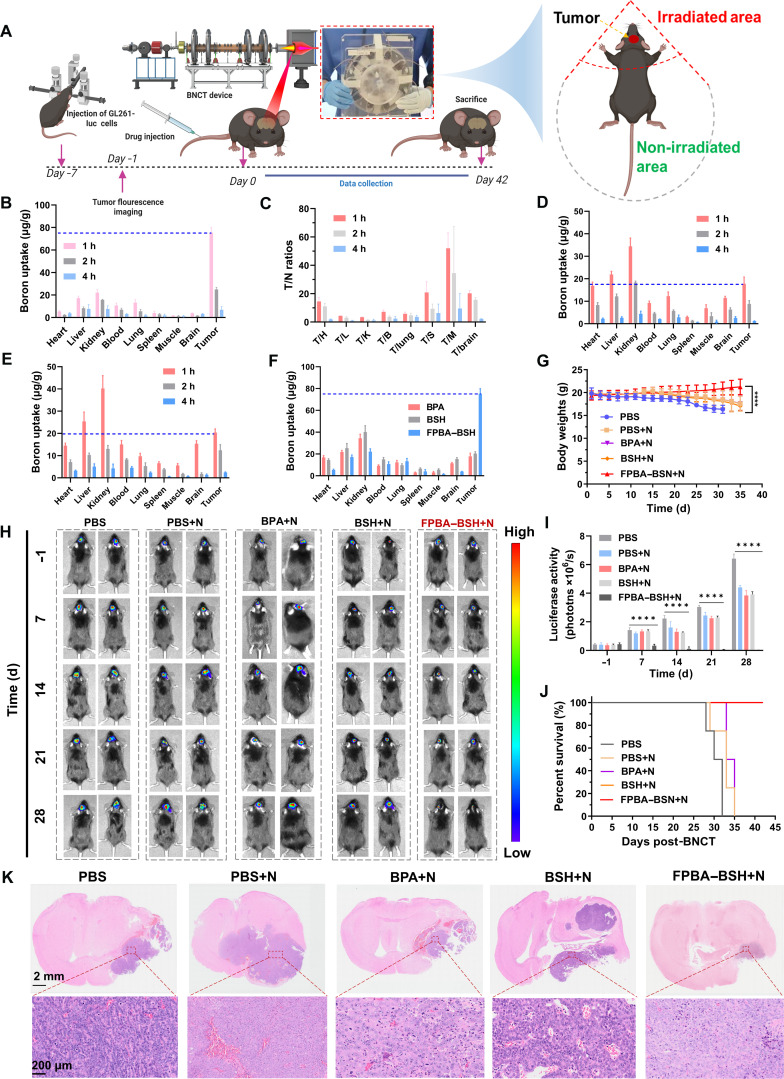
FPBA–BSH in BNCT for brain glioma. (A) Schematic illustration of the antitumor mechanism of BNCT utilizing the FPBA–BSH boron compound. Photograph depicts mice secured in a custom-designed mold for precise neutron irradiation. The red dashed line delineates tissues within the radiation field, while the area between the blue and gray dashed lines marks the non-irradiated zone. (B) Boron concentrations measured in major organs and tumor tissues at 1, 2, and 4 h post-intravenous injection of FPBA–BSH (300 mg/kg) (mean ± SD, *n* = 3). (C) T/N ratios corresponding to data in panel B (mean ± SD, *n* = 3). (D) Boron concentrations in major organs and tumor tissues at 1, 2, and 4 h post-intravenous injection of BPA (mean ± SD, *n* = 3). (E) Boron concentrations in major organs and tumor tissues at 1, 2, and 4 h post-intravenous injection of BSH (mean ± SD, *n* = 3). (F) Boron concentrations in major organs and tumor tissues at 1 h post-intravenous injection of BPA, BSH, and FPBA–BSH (mean ± SD, *n* = 3). (G) Body weight changes in mice subjected to different treatment regimens, monitored over the study period. (H and I) Longitudinal bioluminescence imaging and quantitative analysis of intracranial tumor burden in the glioma-bearing mice at multiple time points following BNCT treatment. (J) Kaplan–Meier survival curves for mice in each treatment group, assessed at designated intervals post-BNCT. (K) H&E staining in mouse brain tissue after BNCT. ****P < 0.0001, *n* ≥ 3.

To assess the therapeutic efficacy of FPBA–BSH in BNCT for brain glioma, we established an orthotopic glioma model by implanting luciferase-expressing GL261 cells into mice. A week post-implantation, the spatial positioning of critical tissues within the neutron irradiation field was carefully mapped to ensure accurate targeting of the brain tumor (Fig. 8A). FPBA–BSH was delivered via intravenous injection at a dose of 300 mg/kg, followed by quantification of boron levels in various organs and tumor tissues that were quantified at multiple time points via ICP-MS. The highest boron accumulation was observed in tumor tissue, reaching an average B concentration of approximately 75.4 μg/g at 1 h postinjection (Fig. 8B), satisfying clinical criteria for effective BNCT. The tumor boron concentration corresponded to an estimated boron dose of approximately 8.1 Gy, yielding a CBE of ~5.8. The ratios of T/N and tumor to blood (T/B) were 52.0 and 7.2, respectively, which exceed the commonly accepted threshold of 3 versus the lungs, heart, brain, and muscle, indicating a favorable biodistribution with minimized off-target radiation risk (Fig. 8C). Subsequently, the in vivo boron biodistribution of BPA and BSH was assessed. As shown in Fig. 8D, BPA achieved a tumor boron concentration of 17.8 μg/g at 1 h postinjection, with T/N and T/B ratios of 2.7 and 1.9, respectively (Fig. S17A). BSH achieved a tumor boron concentration of 20.3 μg/g at 1 h postinjection (Fig. 8E), with T/N and T/B ratios of 3.7 and 1.4 (Fig. S17B). In contrast, FPBA–BSH showed markedly enhanced tumor accumulation. As shown in Fig. 8F, the tumor boron level in the FPBA–BSH group was 4.2- and 3.7-fold higher than that observed for BPA and BSH, respectively. Correspondingly, the T/N and T/B ratios were significantly increased (Fig. S17C). Notably, the T/N ratio of FPBA–BSH was approximately 19.3- and 14.1-fold higher than those of BPA and BSH, respectively, indicating improved tumor enrichment and selectivity relative to the clinically used boron agents.

Notably, body weight decreased in the PBS, PBS+N, BPA+N, and BSH+N groups but increased significantly in the FPBA–BSH+N group (Fig. 8G). Tumor progression monitored by in vivo bioluminescence imaging revealed rapid tumor growth in the PBS group, partial suppression in the PBS+N, BPA+N, and BSH+N groups, and pronounced tumor inhibition in the FPBA–BSH+N group (Fig. 8H and I). Survival analysis demonstrated that 100% of mice receiving FPBA–BSH+N survived beyond 45 d, whereas PBS and PBS+N, BPA+N, and BSH+N groups exhibited median survivals of approximately 30 and 35 d, respectively (Fig. 8J). Immunohistochemical analysis of brain tissues from FPBA–BSH-treated mice subjected to neutron irradiation revealed a marked suppression of tumor cell proliferation, indicated by reduced Ki-67 staining, alongside enhanced apoptosis, evidenced by elevated cleaved Caspase-3 levels (Fig. 8K and Fig. S18A to C). In parallel, histopathological examination by H&E staining revealed no detectable morphological abnormalities in key organs, including the heart, liver, spleen, lungs, and kidneys, relative to controls (Fig. S19).

Together, these data demonstrate that FPBA–BSH effectively augments BNCT-induced tumor eradication in an orthotopic glioma model while maintaining a favorable safety profile.

## Conclusion

In summary, we developed FPBA–BSH, a water-soluble small-molecule ^10^B-enriched carrier with a boron content of 25 wt.%. FPBA–BSH demonstrated excellent biocompatibility and efficient BBB penetration, achieving rapid and selective tumor accumulation. One hour postinjection, tumor boron concentration reached 75.4 μg/g, corresponding to approximately 4.2- and 3.7-fold higher accumulation than BPA and BSH, respectively. The T/N ratio was 52.0, approximately 19.3- and 14.1-fold higher than BPA and BSH, respectively, and the T/B ratio was 7.2, representing 3.8- and 5.1-fold increases over BPA and BSH. These uptake and selectivity metrics meet or exceed clinical benchmarks for BNCT. Under thermal neutron irradiation, FPBA–BSH-mediated BNCT induced pronounced localized cytotoxicity and apoptosis in orthotopic glioma and melanoma models, leading to substantial tumor regression without detectable off-target toxicity. Collectively, these findings support FPBA–BSH as a promising candidate for further preclinical development and potential clinical translation in BNCT.

## Materials and Methods

### Materials

4-Carboxy-3-fluorophenylboronic acid, *N*-(2-aminoethyl) maleimide hydrochloride (MAL-NH_2_), *N,N,N′,N′*-tetramethyl-*O*-(7-azabenzotriazol-1-yl)urea hexafluorophosphate (HATU), *N,N-*diisopropylethylamine, dimethylformamide, methanol (CH_3_OH), 1-octanol, dichloromethane (CH_2_Cl_2_), and trifluoroacetic acid (TFA) were purchased from McLean Chemical Reagent Co. BSH was purchased from Katchem spol. s r. (Czech Republic). 4-Borono-L-phenylalanine (BPA) was purchased from Hainan Poly Pharm Co., Ltd. All chemical reagents were used directly without further purification. PBS, RPMI 1640 cell culture medium, high-glucose Dulbecco’s Modified Eagle Medium (DMEM), fetal bovine serum (FBS), and penicillin–streptomycin were obtained from Gibco. Cell Counting Kit-8 (CCK-8), 4,6-diamidino-2-phenylindole, and Calcein-AM were purchased from Beyotime (China). Dimethyl sulfoxide (DMSO), H&E staining kit, and reduced glutathione content assay kit were purchased from Solarbio (Beijing, China). Live/dead cell staining reagent and Annexin V–fluorescein isothiocyanate (FITC)/PI apoptosis detection kit were purchased from 4A Biotech (Suzhou, China). Deionized water was always used throughout the experimental procedure. All glassware were purchased from Synthware (Chongqing, China). All siRNAs were synthesized by Beijing Tsingke Biotech Co., Ltd (China). The sequences are as follows: Neu3: F 5′-TTCATTGAGCCCCAGGTGAC-3′, R 5′-CAAAGGCCGGAAGCTCACTA-3′; St3gal3: F5′-GTCTCTGGGGTCACGGATTG-3′, R 5′-CAGGGTAGGTGATGCGTAGG-3′. BAY-876 and 3Fax-Neu5Ac were purchased from MedChemExpress (China). siGLUT1-1, siGLUT1-3, and siGLUT1-5 were purchased from Suzhou Ribo Life Science Co., Ltd. Lipomaster 3000 Transfection Reagent was purchased from Vazyme Biotech Co., Ltd.

### Cells and animals

B16F10 cells (Research Resource Identifier [RRID]: CVCL 0159) were purchased from Procell Life Science & Technology Co., Ltd, Wuhan, China. GL261 cells (RRID: CVCL Y003) and GL261-luc cells (RRID: CVCL C9CB) were purchased from the Type Culture Collection, Chinese Academy of Sciences, Shanghai, China. Mouse brain microvascular endothelial cells (bEnd.3) were all purchased from the National Collection of Authenticated Cell Cultures (China). B16F10 cells were cultured in 1640 medium supplemented with 10% FBS (TransSerum FQ Fetal Bovine Serum, China) and 1% penicillin–streptomycin. All cells were cultured in a humidified environment of 37 °C and 5% CO_2_. bEnd.3 cells, GL261 cells, and GL261-luc cells were cultured in high-glucose DMEM supplemented with 10% FBS (TransSerum FQ Fetal Bovine Serum) and 1% penicillin–streptomycin. All cells were cultured in a humidified environment of 37 °C and 5% CO_2_. Female C57BL/6J (4 wk) were purchased from Tengke Biotechnology Co. (Guangzhou, China). All animal experiments were performed in compliance with protocols approved by the Ethics Committee of the Animal Experimentation Institution of The Tenth Affiliated Hospital of Southern Medical University (Dongguan People’s Hospital).

### Synthesis of FPBA–BSH boron drug

4-Carboxy-3-fluorophenylboronic acid (1.2 mmol) was activated with HATU (1.2 mmol) and *N,N-*diisopropylethylamine (6.0 mmol) in dimethylformamide (10 ml) at room temperature for 30 min, after which *N*-(2-aminoethyl)maleimide hydrochloride (1.0 mmol) was added. The reaction was stirred for 5 h and monitored by thin-layer chromatography. After solvent removal under reduced pressure, the crude material was purified through silica gel chromatography using a dichloromethane/methanol (MeOH) (10:1) solvent system to afford 3-fluoro-4-(2-(maleimido)ethylcarbamoyl)phenylboronic acid (FPBA-MAL) was obtained as a white solid in 90% yield. ^1^H NMR (600 MHz, DMSO-d6): δ 8.42 to 8.38 (m, 1H), 7.62 (d, *J* = 7.5 Hz, 1H), 7.54 (d, *J* = 11.2 Hz, 1H), 7.49 to 7.46 (m, 1H), 7.01 (s, 2H), 3.63 to 3.60 (m, 1H), 3.59 to 3.56 (m, 2H), 3.42 to 3.38 (m, 2H), and 3.16 to 3.12 (m, 1H). Electrospray ionization (ESI)/MS (*m/z*) calcd for C_13_H_12_BFN_2_O_5_ [M+H]^+^: 307.0903; found: 307.0904.

FPBA-MAL (1.0 mmol) was dissolved in methanol under a nitrogen atmosphere, followed by the introduction of ^10^BSH (1.0 mmol) into the reaction. The reaction system was stirred at room temperature for 24 h and subsequently purified by preparative high-performance liquid chromatography (0.1% TFA in H_2_O/0.1% TFA in MeOH = 75:25, *v/v*). The product was isolated as a white solid following lyophilization, affording FPBA–BSH in 70% yield. ESI/MS (*m/z*) (ESI): calcd for C_13_H_24_B_13_FN_2_O_5_S [M+Na]^+^: 492.2853; found: 492.2855. Purity: 100.00% (220 nm).

### Water solubility analysis

In this work, the Log *P* of the compound was measured using the shake-flask method to evaluate its lipophilicity. A known quantity of the compound was dissolved in a defined volume of octanol (oil phase) and an aqueous buffer solution (water phase) in a separatory funnel, and the reaction mixture was vigorously agitated for 30 min at 25 °C to ensure equilibrium. After a stabilization period of 1 h, the phases were allowed to separate completely. The boron concentrations of the compound present in the aqueous phase and the organic phase were determined by ICP-MS. The partition coefficient was then calculated using the formula Log *P* = log ([*C*_octanol_] / [*C*_water_]), where [*C*_octanol_] and [*C*_water_] represent the levels of the compound in the octanol and aqueous layers, respectively.

### Cell viability assay

Cell viability was assessed using a CCK-8 assay. For evaluation of FPBA-BSH cytotoxicity, B16F10 and GL261 cells were seeded in 96-well plates at 1 × 10^3^ cells per well and cultured overnight in RPMI-1640 medium supplemented with 10% FBS and 100 U/ml penicillin/streptomycin at 37°C in a humidified atmosphere containing 5%CO_2_. The medium was then replaced with fresh medium containing FPBA-BSH at boron concentrations of 0, 10, 25, 40, 50, or 100 µg/ml. After incubation for 24 or 48 h, 10 µl of CCK-8 reagent was added to each well, followed by incubation for 3 h. Absorbance at 450 nm was measured using a microplate reader. Untreated cells served as the control, and wells containing medium alone served as blanks. Cell viability was calculated as (*A*_sample_ − *A*_blank_)/(*A*_control_ − *A*_blank_) × 100%. Data are presented as mean ± SD (*n* = 5).

For BAY-876 treatment, B16F10 and GL261 cells were seeded in 96-well plates at 1 × 10^3^ cells per well, allowed to attach overnight, and then treated with BAY-876 at serial concentrations ranging from 0.1 nM to 2 µM (10-point log10 dilution series) or with DMSO as the vehicle control. After 72 h of incubation, 10 µl of CCK-8 reagent was added to each well and the plates were incubated for an additional 2 h at 37°C. Absorbance at 450 nm was recorded using a Tecan Spark microplate reader. Cell viability was normalized to the DMSO-treated group, which was defined as 100%. Each condition was tested in six technical replicates.

For 3Fax-Neu5Ac treatment, B16F10 and GL261 cells were seeded in 96-well plates at 1 × 10^3^ cells per well and cultured overnight, followed by treatment with 3Fax-Neu5Ac at 0.1, 1, or 10 µM for 48 h. Untreated and vehicle-treated cells were included as controls. After treatment, 10 µl of CCK-8 reagent was added to each well and incubated for 2 h at 37°C, and absorbance at 450 nm was measured using a Tecan Spark microplate reader. Cell viability was normalized to the corresponding control group, which was set to 100%. Each condition was analyzed in six technical replicates.

### Cell scratch assay

Approximately 1 × 10^5^ cells were inoculated into each well of a 6-well plate and cultured in complete RPMI 1640 medium containing 10% FBS until they reached 80% fitness. Subsequently, the cells were exposed to different boron concentrations of FPBA–BSH (10, 25, 40, 50, and 100 μg/ml) for 24 h. Scratches were made with the tip of a 20-μl pipette needle at 0.5-cm intervals. Cells were then washed 3 times with PBS to remove any displaced cells and replaced with serum-free RPMI 1640. Cultures were stored at 37 °C, 5% CO_2_. Micrographs were taken with an inverted phase contrast microscope at 10× magnification, and the gap-filling area between the 3 time points of 0, 24, and 48 h was calculated using ImageJ software.

### Hemolysis experiment

One milliliter of fresh blood from mice was taken and centrifuged to separate the blood cells (1,500 rpm, 15 min, 4 °C). Erythrocytes were washed 5 times with saline until the supernatant became clear and then resuspended with saline to prepare 2% erythrocyte suspension. PBS was used as the negative control, and distilled water was used as the positive control. Blood cells (0.2 ml) were added to the PBS solution containing different boron concentrations of FPBA–BSH (10, 25, 40, 50, and 100 μg/ml) and incubated for 3 h. The blood samples were centrifuged at high speed (5,000 rpm, 15 min, 4 °C), and the absorbance (*A*) of the supernatant at wavelength 540 nm was calculated. The hemolysis rate of each sample was determined using the following formula: hemolysis rate (%) = (Sample − Negative) / (Positive − Negative) × 100%.

### Quantitative real-time PCR

B16F10 and GL261 cells were seeded into 6-well plates at a density of 8 × 10^5^ cells per well and exposed to 3Fax (10 μM) or vehicle control (DMSO) for 48 h. Total RNA was extracted with the RNeasy Mini Kit (Qiagen) following the manufacturer’s protocol. RNA purity and integrity were evaluated using a NanoDrop 2000 spectrophotometer (*A*_260_/*A*_280_ ratio of 1.8 to 2.0) and confirmed by agarose gel electrophoresis. Subsequently, 1 μg of total RNA was reverse transcribed into cDNA using the iScript Reverse Transcription Supermix (Bio-Rad) in a 20-μl reaction, with the following cycling conditions: 25 °C for 5 min, 46 °C for 20 min, and 95 °C for 1 min. qPCR reactions (20 μl total volume) consisted of 10 μl SsoAdvanced Universal SYBR Green Supermix (Bio-Rad), 1 μl of diluted cDNA template (1:10), and 0.5 μM gene-specific primers (Neu3: F 5′-TTCATTGAGCCCCAGGTGAC-3′, R 5′-CAAAGGCCGGAAGCTCACTA-3′; St3gal3: F 5′-GTCTCTGGGGTCACGGATTG-3′, R 5′-CAGGGTAGGTGATGCGTAGG-3′). qPCR amplification was performed on a CFX96 Real-Time PCR Detection System (Bio-Rad) using the following cycling protocol: 95 °C for 3 min, followed by 40 cycles of 95 °C for 10 s and 60 °C for 30 s. Melting curve analysis (65 to 95 °C) was conducted to verify amplicon specificity.

B16F10 and GL261 cells were seeded at 5.0 × 10^5^ cells per well in 6-well plates and cultured for 18 to 24 h prior to transfection. Cells were transfected with GLUT1 siRNA or scrambled control siRNA using siLentFect at a final siRNA concentration of 50 nM. siRNA–lipid complexes were prepared in serum-free RPMI 1640 or DMEM by incubation for 20 min at room temperature and added to cells for 4 h, followed by replacement with complete medium containing 10% FBS. Knockdown efficiency was evaluated by qPCR at 24 h, and functional assays were conducted 48 h after transfection.

### Molecular docking

In the molecular docking experiment to assess the interaction between small molecules and proteins, the process begins with the preparation of the protein structure, which is optimized and refined using Discovery Studio 2019 software to ensure accurate binding site representation. Subsequently, the small molecules are prepared and docked against the target protein using the software’s docking algorithms, allowing for the prediction of binding affinities and conformational poses. Following the docking procedure, the results are analyzed and visualized using PyMOL software, which facilitates the examination of the binding interactions through detailed structural representations and molecular interactions.

### 2-NBDG glucose uptake assay by flow cytometry

Log-phase B16F10/GL261 cells were plated in 6-well plates at 5 × 10^5^ cells per well and incubated overnight to allow adhesion. Cells were subsequently treated with BAY-876 (10 μM) or an equivalent volume of DMSO (vehicle control) for 24 h. After treatment, cells were rinsed twice with RPMI 1640/glucose-free DMEM and exposed to 100 μM 2-deoxy-2-[(7-nitro-2,1,3-benzoxadiazol-4-yl)amino]-D-glucose (2-NBDG; Thermo Fisher Scientific) prepared in RPMI 1640/glucose-free DMEM at 37 °C for 30 min.

For staining and acquisition, cells were detached using 0.05% trypsin–EDTA, washed twice with FACS buffer (PBS supplemented with 2% FBS), and resuspended at 1 × 10^6^ cells/ml. 2-NBDG fluorescence was measured on a CytoFLEX flow cytometer (Beckman Coulter) fitted with a 488-nm excitation laser and a 530/30-nm emission filter. Viable single cells were gated according to forward and side scatter parameters, with 10,000 events collected per sample. Fluorescence intensity histograms were generated using CytExpert software (v2.4). The relative glucose uptake capacity was quantified by comparing the shift in 2-NBDG fluorescence between BAY-876-treated and DMSO control cells.

### *In vitro* and *in vivo* uptake of FPBA–BSH

The FPBA–BSH boron drug with boron concentrations of 0, 10, 25, 40, 50, and 100 μg/ml was allowed to co-cultivate with the cells for 0.5, 1, 2, and 4 h, then the medium was subsequently removed, and the cells were washed 3 times with PBS, and then the boron concentration in 1 × 10^6^ cells was measured by ICP-MS. Boron drugs (300 mg/kg) were administered to tumor-bearing mice via the tail vein, after which the animals were euthanized at 1, 2, or 4 h after administration, and the tumors and organs were collected. The collected samples were soaked in 1 ml of 70% nitric acid, and the mixture was heated at 70 °C for 2 h. The incinerated samples were diluted with 1% nitric acid and adjusted to a volume of 5 ml. The samples were filtered through a syringe filter (0.22 μm) and then quantified for boron content using ICP-MS (Agilent 7900 ICP-MS, USA).

### RNA-seq analysis

The RNA-seq analysis was performed by LC-Bio Technology Co., Ltd. (Hangzhou, China). Samples were sent to LC-Bio to perform RNA extraction and purification, reverse transcription, sequencing, and library construction. Total RNA was extracted using TRIzol reagent (Thermo Fisher, 15596018). RNA quantity and purity were assessed using a Qubit 3.0 fluorometer (Thermo Fisher, Q33216) and an Agilent 5300 Fragment Analyzer (Agilent, M5311AA). The transcriptome library for RNA-seq was created using 2.0 μg of total RNA (RIN > 7.0) with mRNA Capture Beads 2.0 (Yeasen, 12629ES) and the Hieff NGS Ultima Dual-mode mRNA Library Prep Kit (Yeasen, 12340ES97) according to the manufacturer’s protocol. Library construction included mRNA purification, fragmentation, first-strand and second-strand cDNA synthesis, end repair, A-tailing, adapter ligation, and PCR amplification. The final libraries with an average insert size of 400 ± 50 bp were sequenced on an Illumina NovaSeq X Plus platform using the PE150 strategy. After quality control of the raw reads, the clean reads were mapped using HISAT2 software and further assembled by StringTie.

### Colony formation experiment

The growth potential of cancer cells after different treatments was evaluated by colony formation ability. The cells were divided into PBS group, PBS plus neutron irradiation group, FPBA–BSH group, and FPBA–BSH irradiation group, then the PBS plus neutron irradiation group and FPBA–BSH irradiation group were subjected to neutron irradiation for 10 min, and then the cells were incubated with a new medium for 14 d and then stained with 0.25% crystal violet for about 30 min. The above experiments were repeated 3 times. The survival rate was calculated according to the standard method. Cells exposed to culture medium alone served as the control. Data were analyzed using ImageJ and are presented as a comparison with the number of untreated colonies.

### Apoptosis analysis

Cell death was assessed using the Annexin V–FITC Apoptosis Detection Kit. The cells were divided into PBS group, PBS plus neutron irradiation group, FPBA–BSH group, and FPBA–BSH irradiation group, then the PBS plus neutron irradiation group and FPBA–BSH irradiation group were subjected to neutron irradiation for 10 min, and then the cells were inoculated into 6-well plates with a density of 1 × 10^5^ per well and incubated at 37 °C for 48 h. Following initial culture, cells were exposed to neutron irradiation or left untreated. The cells were then incubated for an additional 24 h, and the cells were stained with propidium iodide and membrane-bound protein V following the manufacturer’s instructions for the apoptosis detection kit and then analyzed by flow cytometry on a Fluorescence-Activated Cell Sorting (FACS) Canto II system. Cells treated only with culture medium were designated as the control group.

### Live/dead staining method

The cells were divided into PBS group, PBS plus neutron irradiation group, FPBA–BSH group, and FPBA–BSH irradiation group, and then the PBS plus neutron irradiation group and FPBA–BSH irradiation group were subjected to neutron irradiation for 10 min and then seeded in 6-well plates at 1.0 × 10^5^ cells per well, followed by incubation for 48 h. Cells were subsequently visualized using a fluorescence inverted microscope following the manufacturer’s instructions for the Calcein-AM/PI double staining kit.

### γH2AX immunofluorescence staining

After various treatments, GL261 cells were fixed with 4% paraformaldehyde, permeabilized, blocked, and incubated with anti-γH2AX antibody (Abcam, ab81299) overnight at 4 °C. After washing, the cells were incubated with the Alexa Fluor 555-conjugated secondary antibody (Abcam, ab150078) for 1 h at room temperature. Finally, DAPI was used to label the cell nuclei. The γH2AX foci in the cells were visualized using CLSM.

### *In vitro* BBB permeability assay

An in vitro experiment BBB model was developed using a 24-well Transwell insert system (0.4 μm pore size; Corning). bEnd.3 endothelial cells were cultured at a seeding density of 1 × 10^5^ cells/cm^2^ in the upper chamber and cultured until they reached 90% confluence. The lower compartment was loaded with DMEM/F-12 medium supplemented with 1% FBS to maintain cell growth and viability. During the culture period, transendothelial electrical resistance was measured regularly to assess the formation of the BBB. A stable transendothelial electrical resistance value within the range of 200 to 600 Ω·cm^2^ was considered indicative of successful BBB formation. Following the establishment of the model, the drug was dissolved in PBS and introduced into the upper chamber. The drug was exposed for 1 h. After the exposure period, media from the lower chamber were collected. The concentration of boron (B) in the samples was quantified by ICP-MS, allowing the assessment of the drug’s permeability across the BBB.

### Animal experiment and tumor model

Female C57BL/6J mice were obtained from Tengke Biotechnology Co. Mice at 4 to 6 weeks of age and weighing more than 15 g were selected. All animal experiments were conducted in accordance with protocols approved by the Institutional Animal Care and Use Committee of The Tenth Affiliated Hospital of Southern Medical University (Dongguan People’s Hospital) (No. SL202507014). To establish the tumor model, mice were housed under specific pathogen-free conditions in accordance with protocols approved by the institutional ethics committee. After 1 week of acclimatization, 5 × 10^6^ B16F10 cells in 100 μl of serum-free medium were injected subcutaneously into the outer thighs of mice. Subcutaneous tumor growth was monitored every 2 d and assessed using a vernier caliper. Follow-up experiments were performed on day 14 after tumor inoculation, and when subcutaneous tumors reached a volume of ~50 mm^3^, the compliant mice were randomly assigned to PBS, PBS+N, BPA+N, BSH+N, and FPBA–BSH+N groups with *n* = 6 in each group. Intravenous injection of BPA, BSH, and FPBA–BSH boron drug (300 mg/kg) for 1 h was followed by BNCT treatment. We measured the tumor diameter with calipers every 2 d and assessed the tumor volume according to the formula *V* = (length × width^2^)/2. Body weight was monitored for each mouse at 2-d intervals. After 14 d of treatment, some mice were euthanized.

Establishment of an orthotopic glioma mouse model: Eight-week-old female C57BL/6J mice (≈18 g) were anesthetized with 2% sodium pentobarbital and had their heads shaved. An orthotopic glioma model was established by stereotactic injection of 1 × 10^5^ GL261-luc cells in 5 μl sterile PBS into the right corpus striatum (1.8 mm lateral, 0.6 mm anterior to bregma, and 3.0 mm depth) using a mouse adaptor. The orthotopic glioma mouse model was randomly divided into 5 groups, PBS, PBS+N, BPA+N, BSH+N, and FPBA–BSH+N groups, *n* = 4 in each group. BNCT was performed after intravenous injection of BPA, BSH, and FPBA–BSH boron (300 mg/kg) for 1 h. Mice were subjected to BNCT for 1 h. Tumor growth was monitored on days 7, 14, 21, and 28 after exposure to irradiation using bioluminescence imaging that was performed with an in vivo imaging system from PerkinElmer USA.

### BNCT irradiation conditions

The neutron irradiation parameters were as follows: the neutron flux was approximately 0.8 × 10^9^ n·cm^−2^·s^−1^, and the irradiation time was 30 min. The total neutron dose (*D*_B_), neutron dose (*D*_n_), neutron relative biological effectiveness (*RBE*_beam_), compound biological effectiveness (*CBE*), and the absorbed dose resulting from the interaction of the neutron beam with the tissue itself (*D*_neutron_) were as follows [32]:

$$D_{B}\;=\;7.43\;\times \;10^{-14}\;\times \;C_{B}\;\times \;\Phi$$ (1)

$$D_{n}\;=\;\Phi \;\times \;3.2\;\times \;10^{-13}$$ (2)

Φ = ∅ × T (3)

$${\text{RBE}}_{\mathrm{beam}}\;=\;\frac{D_{x-\text{ray}}}{D_{\text{neutron}}}$$ (4)

$$\mathrm{CBE}\;=\;\frac{D_{x-\mathrm{ray}}\;-\;D_{\mathrm{beam}}\;\times \;{\text{RBE}}_{\mathrm{beam}}\;}{D_{B}}$$ (5)

$$D_{\text{neutron}}\;=\;D_{\gamma }\;+\;D_{N}\;+\;D_{H}$$ (6)

Here, ∅ = 0.8 × 10^9^ n·cm^−2^·s^−1^, *T* = 1,800 s, $D_{\gamma }$ = 0.5 Gy, *C*_B_ represents the boron concentration in tumor tissues, *D*_x-ray_ = 50 Gy, and *D*_n_ = 0.5 Gy. Substituting these values into the above formula yields the physical dose of boron reaction *D*_B_, *CBE*. In the melanoma model: *C*_B_ = 114.4 ppm, *D*_neutron_ = 13.2 Gy, *D*_B_ = 12.2 Gy, and *CBE* = 3.9. In the glioma model: *C*_B_ = 75.4 ppm, *D*_neutron_ = 9.1 Gy, *D*_B_ = 8.1 Gy, and *CBE* = 5.8.

## Data Availability

Data will be made available on request.
