# Supplementary material for: Small-Molecule Boron-10-Enriched Carriers with Exceptional Aqueous Solubility for Enhanced Boron Neutron Capture Therapy of Malignant Tumors
Source: Research (Wash D C). 2026 Jul 3;9:1315. doi: 10.34133/research.1315 (PMC13329701; doi:10.34133/research.1315)
Supplement: Supplementary 1 — Figs. S1 to S19 Tables S1 to S3 [file research.1315.f1.docx]

**Supplementary Materials**

**Small Molecule Boron-10-Enriched Carriers with Exceptional Aqueous Solubility for Enhanced Boron Neutron Capture Therapy of Malignant Tumors**

***Tongyin Xiong^1,2^, Xiangdi Yang^1^, Tingting Li^1^, Rongtao Song^1,2^, Linxuan Huang^1^, Xueli Sang^1^, Fang Hu^2^*, Xiao Xu^1^*, Zhigang Liu^1^****

^1^Cancer Center, Guangdong Engineering Research Center of Boron Neutron Therapy and Application in Malignant Tumors, Dongguan Key Laboratory of Precision Diagnosis and Treatment for Tumors, The Tenth Affiliated Hospital, Southern Medical University (Dongguan People’s Hospital), Dongguan 523059, China.

^2^ Biomaterials Research Center, School of Biomedical Engineering, Guangdong Provincial Key Laboratory of Medial Image Processing, Southern Medical University, Guangzhou 510515, China*.*

*Address correspondence to: [hufang19@smu.edu.cn](mailto:hufang19@smu.edu.cn) (F. H);

*Address correspondence to: [xiaoxu721@smu.edu.cn](mailto:xiaoxu721@smu.edu.cn) (X. X);

*Address correspondence to: [Zhigangliu1983@hotmail.com](mailto:Zhigangliu1983@hotmail.com) (Z. L);

**Fig. S1-S19**


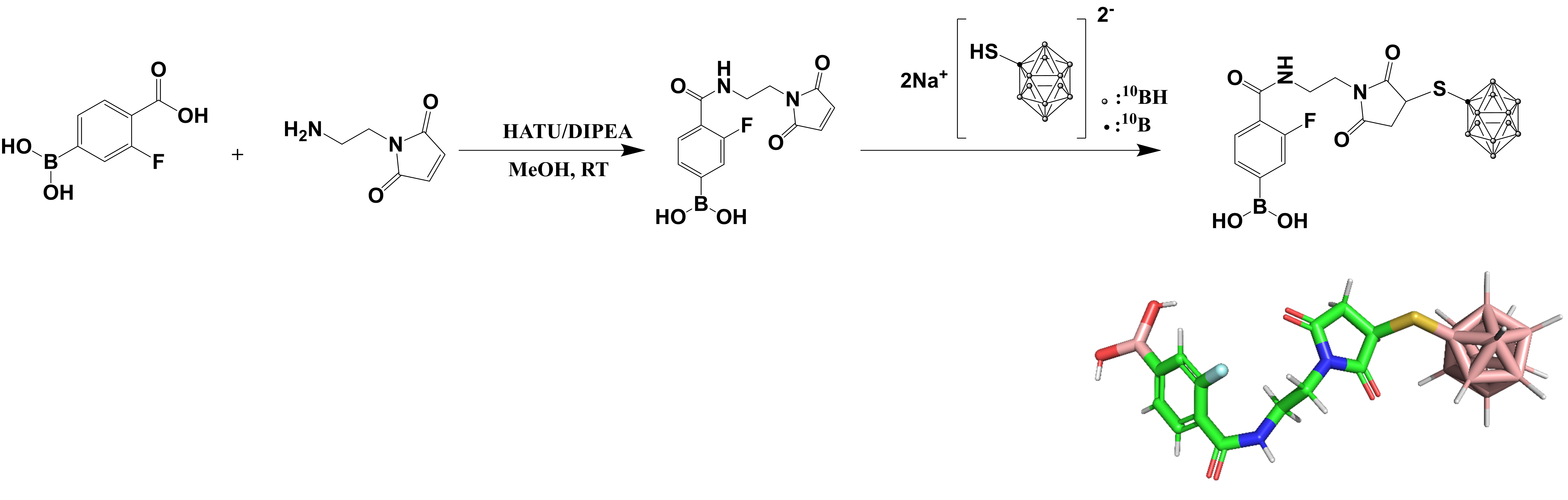


**Fig. S1** FPBA-BSH Compound Synthesis Route and 3D modelling diagram.





**Fig. S2 (A)** ^1^H NMR spectrum of FPBA-MAL in CD_3_OD. **(B)** ESI/MS spectrum of FPBA-MAL.


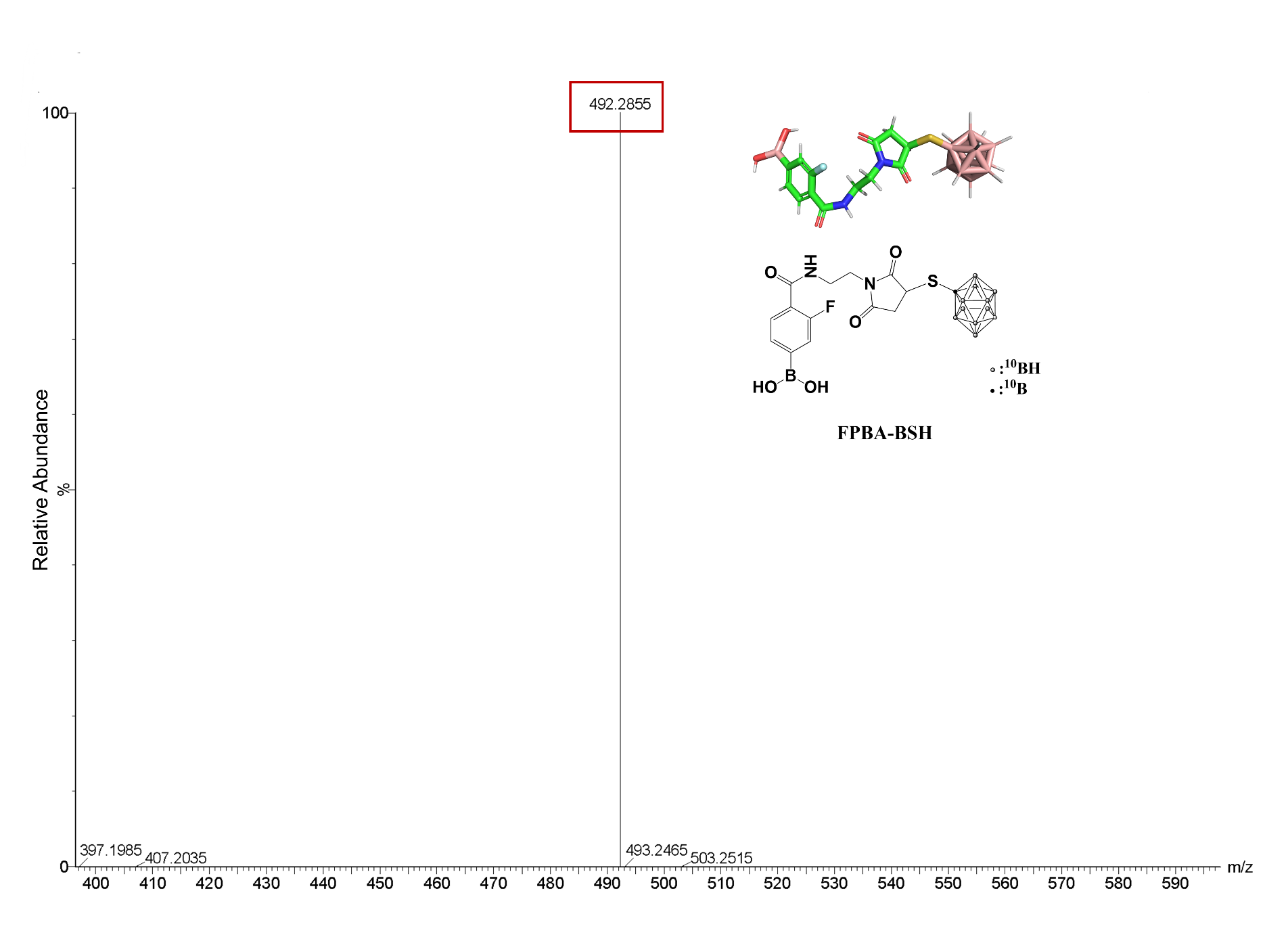


**Fig. S3** ESI/MS spectrum of FPBA-BSH.

**
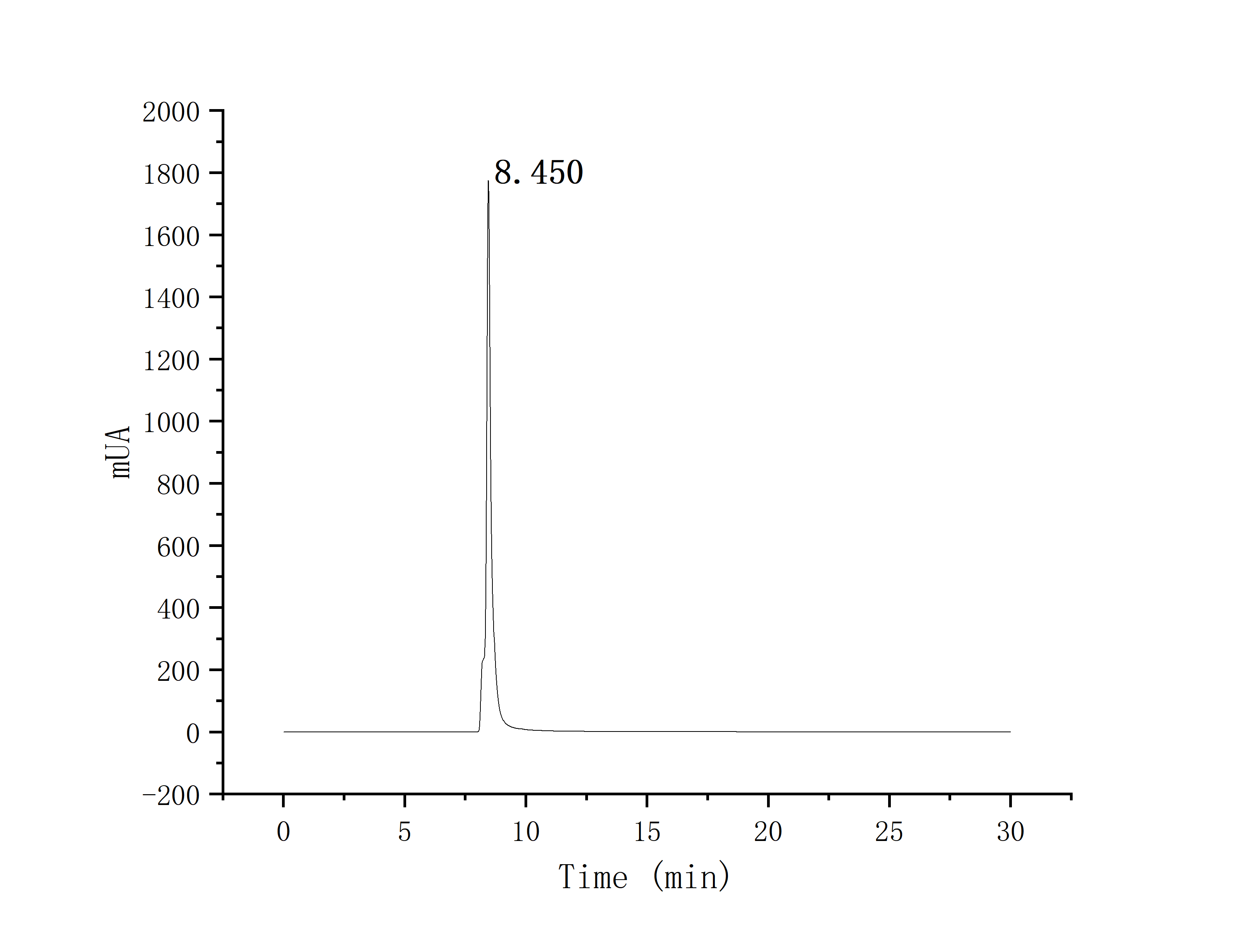
**

| Integration Result | | | | |
| --- | --- | --- | --- | --- |
|  | Retention Time (min) | Peak Area (mAU*min) | Peak Height (mAU) | Relative Peak Area (%) |
|  | 8.450 | 304.629 | 1775.126 | 100.00 |
| Total | | 304.629 | 1775.126 | 100.00 |

**Fig. S4** HPLC spectrum of FPBA-BSH.

**Table S1. The boron content of the boron-based medicine Chart.**

|  | Name | Structural formula | Boron content | DOI |
| --- | --- | --- | --- | --- |
| A | BPA |  | 4.8% | 10.1021/ja01537a021 |
| B | FBY |  | 4.8% | 10.1021/acs.bioconjchem.9b00578 |
| C | BPA-Tyr |  | 2.6% | 10.1016/j.jphs.2019.01.012 |
| D | BTS |  | 4.4% | 10.1021/acs.jmedchem.3c01265 |
| E | BBPA |  | 9.4% | 110.1002/anie.202413249 |
| F | R-^18^F-5a |  | 5% | 10.1021/acs.molpharmaceut.2c00036 |
| G | DPA-BSH |  | 19% | 10.1021/acsmedchemlett.1c00377 |
| H | Crgd-Mid-BSA | 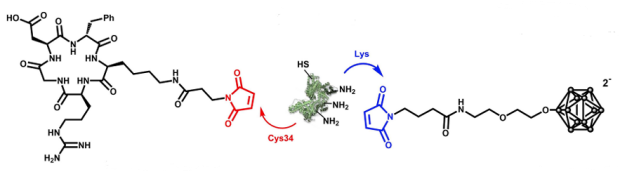 | 3.7% | 10.1021/acs.molpharmaceut.0c00478 |
| I | closo-Dodecaborate-(Ga-DOTA)-c(RGDfK) |  | 8.1% | 10.1021/acs.jmedchem.2c01586 |
| J | 3-BPA |  | 4.8% | 10.3390/pharmaceutics14051106 |
| K | PSMA-1h |  | 17% | 10.1021/acs.molpharmaceut.9b00464 |
|  | FPBA-BSH | 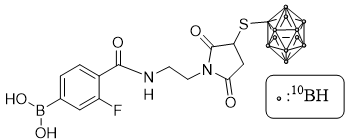 | **25.5%** | **This work** |


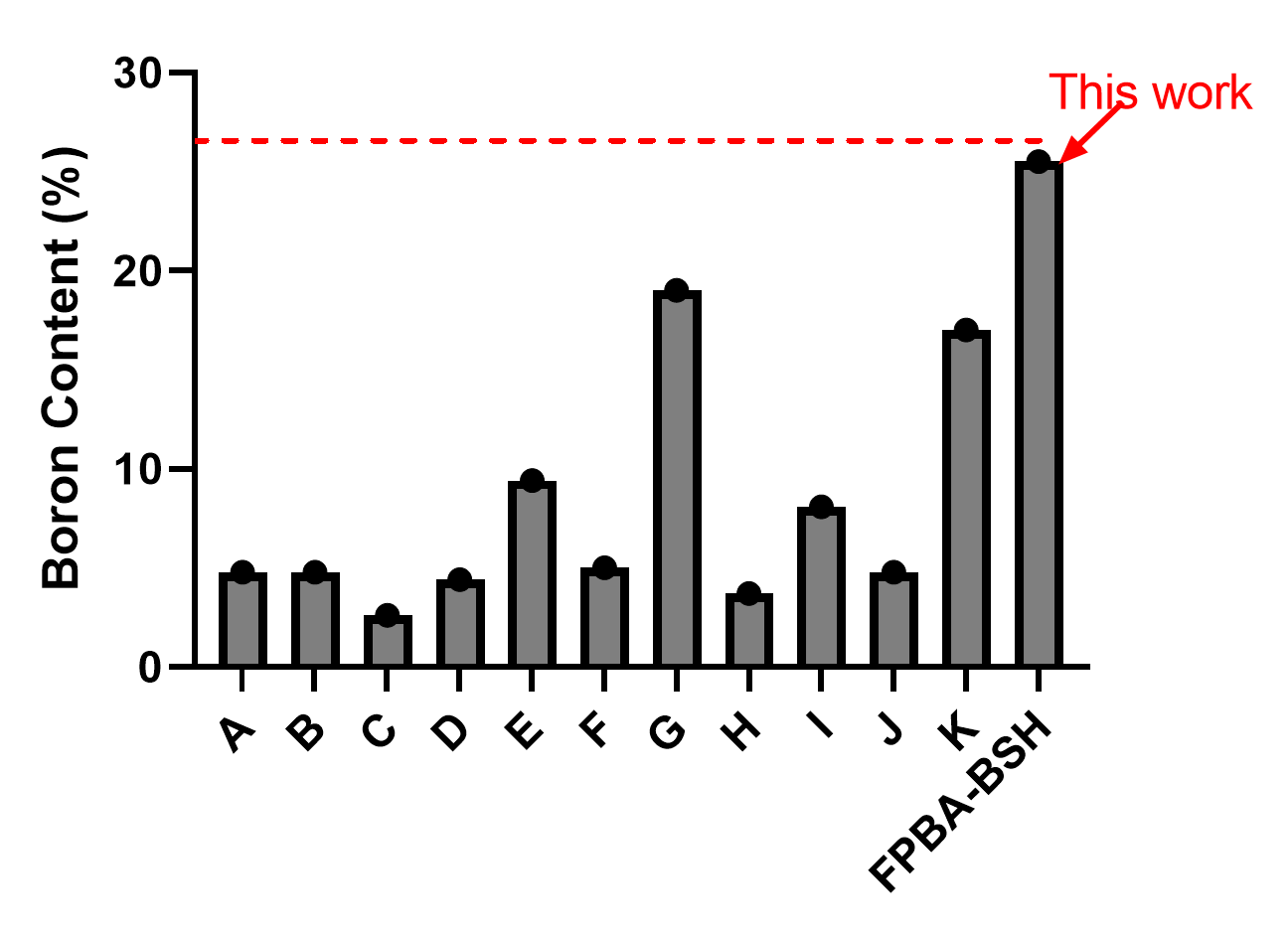


**Fig. S5** Statistical charts showing the proportion of boron drugs in the literature in recent years.


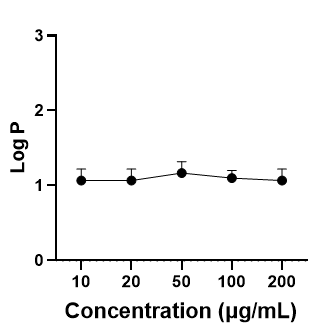


**Fig. S6** FPBA-BSH lipid-water partition coefficient.


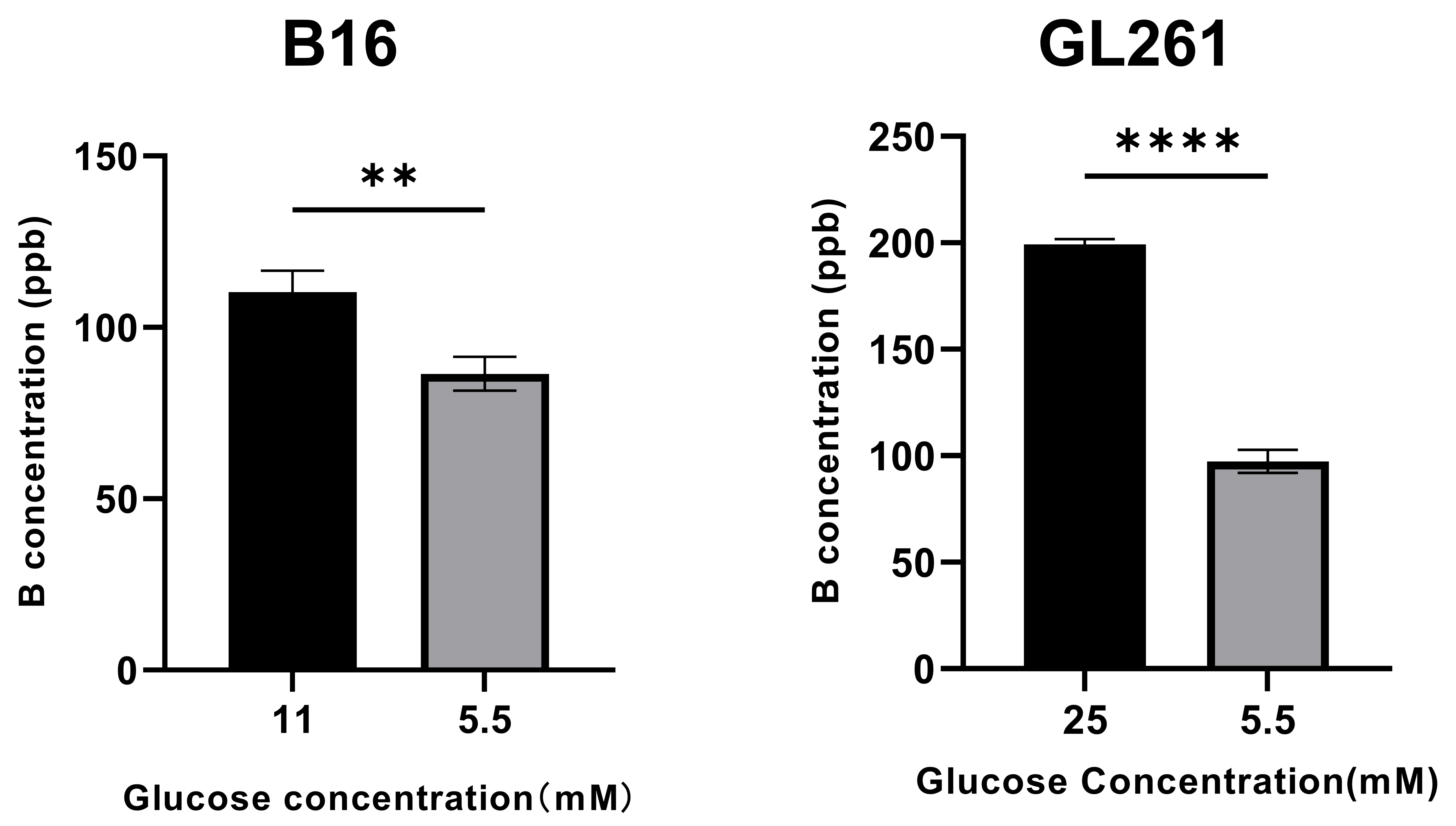


**Fig. S7** Cellular uptake of FPBA-BSH under different extracellular glucose concentrations. ***P* < 0.01, *****P* < 0.0001 (statistical significance based on unpaired two-tailed t-test).


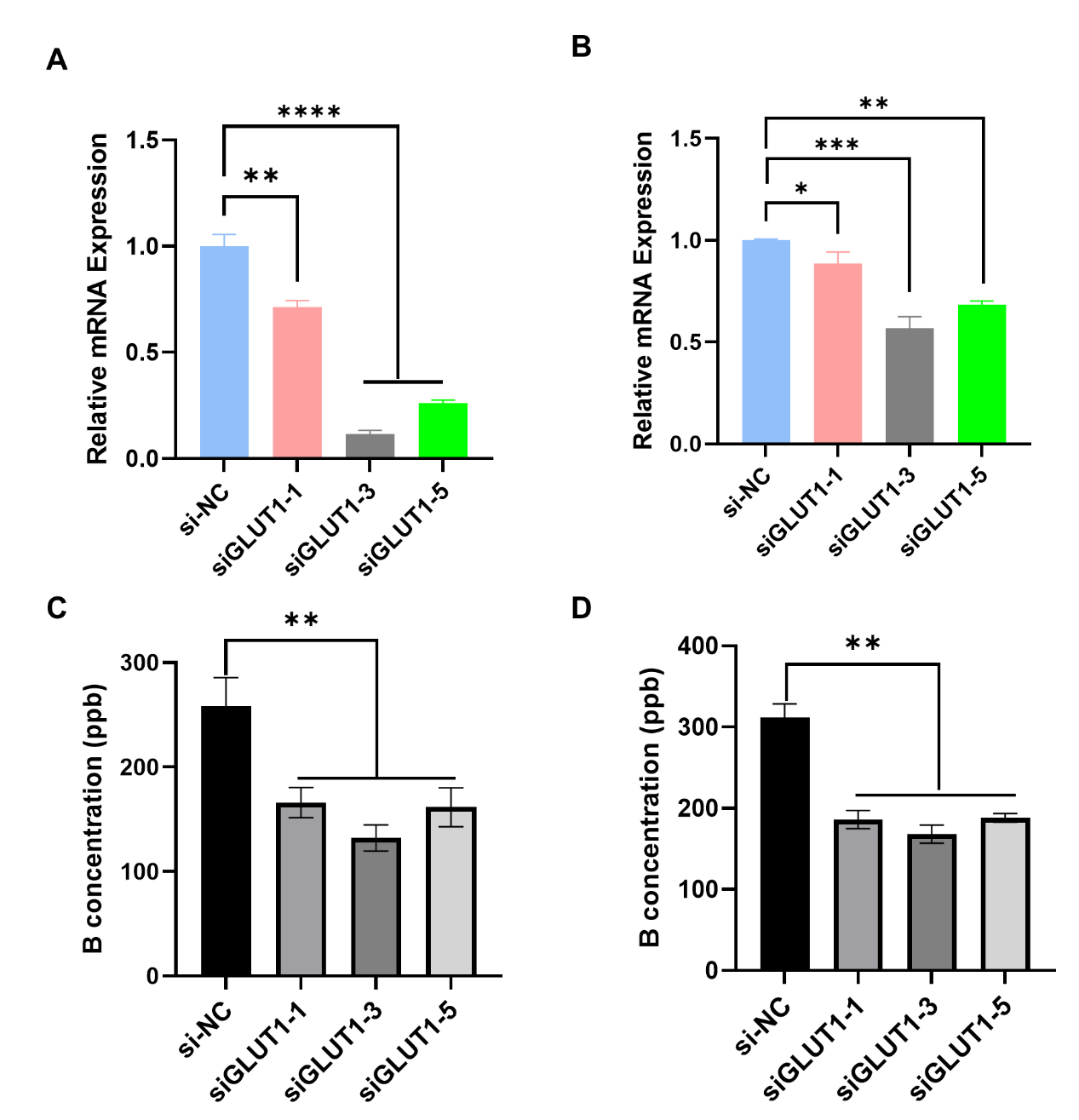


**Fig. S8** (A) qPCR analysis of relative GLUT1 mRNA expression in B16F10 cells after transfection with different GLUT1-targeting siRNAs. (B) qPCR analysis of relative GLUT1 mRNA expression in GL261cells after transfection with different GLUT1-targeting siRNAs. (C) Cellular uptake of FPBA-BSH in B16F10 cells following GLUT1 knockdown by different siRNA treatments. (D) Cellular uptake of FPBA-BSH in GL261cells following GLUT1 knockdown by different siRNA treatments. **P* < 0.1, ***P* < 0.01, ****P* < 0.001, *****P* < 0.0001 (statistical significance based on unpaired two-tailed t-test).

**
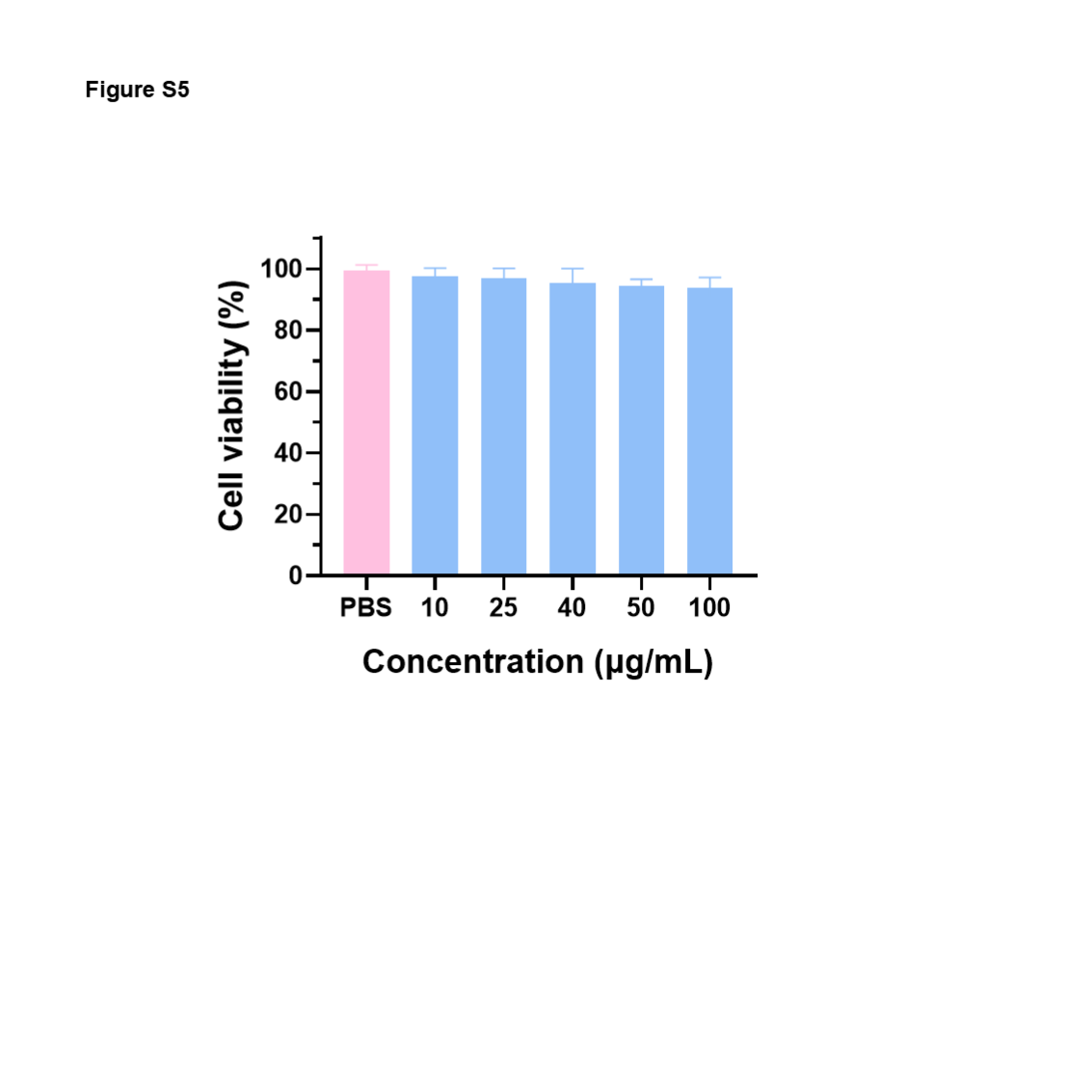
**

**Fig. S9** Relative survival of GL261 cells after incubation with different concentrations of FPBA-BSH for 48h.

**Table S2** Molecular Docking Scoring Chart (A) GLUT1 Molecular Docking Scoring (B) SIGLEC1 Molecular Docking Scoring

**A.**

| Composite mode | Appetency (kcal/mol) | Cluster RMSD | Reference RMSD |
| --- | --- | --- | --- |
| 1 | -7.92 | 0 | 55.3 |
| 2 | -7.69 | 0 | 56.26 |
| 3 | -7.64 | 0 | 57.21 |
| 4 | -7.56 | 0 | 54.14 |
| 5 | -7.56 | 0 | 54.99 |
| 6 | -6.74 | 0 | 54.98 |
| 7 | -6.65 | 0 | 55.87 |
| 8 | -6.53 | 1.87 | 56.27 |
| 9 | -5.96 | 0 | 59.15 |

**B.**

| Composite mode | Appetency (kcal/mol) | Cluster RMSD | Reference RMSD |
| --- | --- | --- | --- |
| 1 | -5.32 | 0 | 18.58 |
| 2 | -4.58 | 0 | 18.49 |
| 3 | -4.33 | 0 | 24.90 |
| 4 | -4.21 | 0 | 23.81 |
| 5 | -3.62 | 0 | 24.30 |
| 6 | -3.48 | 0 | 24.76 |
| 7 | -3.32 | 0 | 12.68 |
| 8 | -2.38 | 0 | 33.48 |
| 9 | -1.72 | 0 | 35.95 |


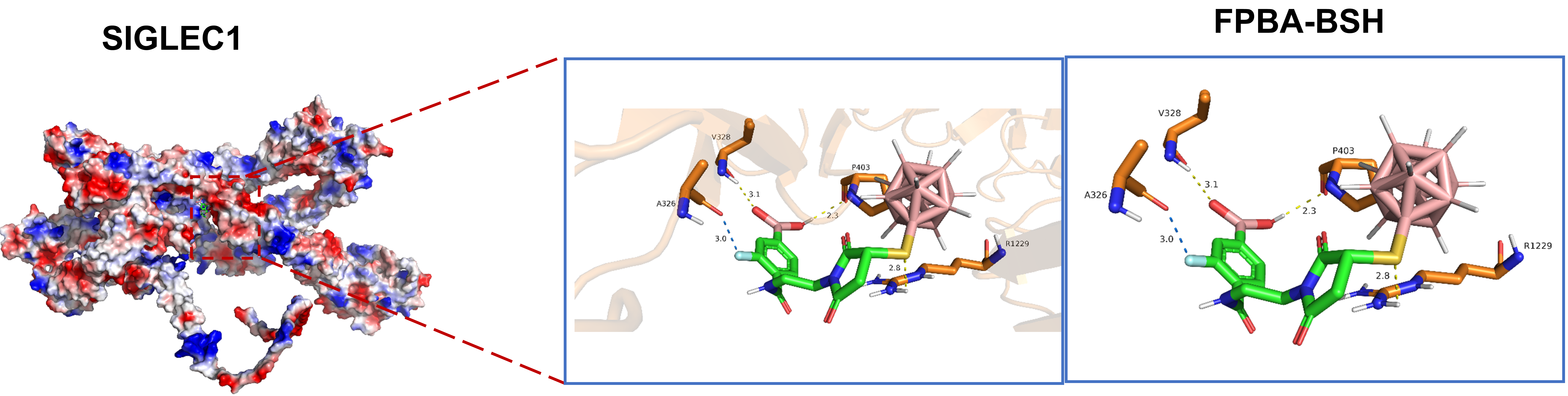


**Fig. S10** Molecular docking simulations illustrating the predicted binding interactions between FPBA-BSH and the Sialic Acid Binding Ig Like Lectin 1（SIGLEC1）, with detailed views of hydrogen-bonding networks.





**Fig.**  **S11** Statistical chart of boron uptake in tumors in recent years in the literature.


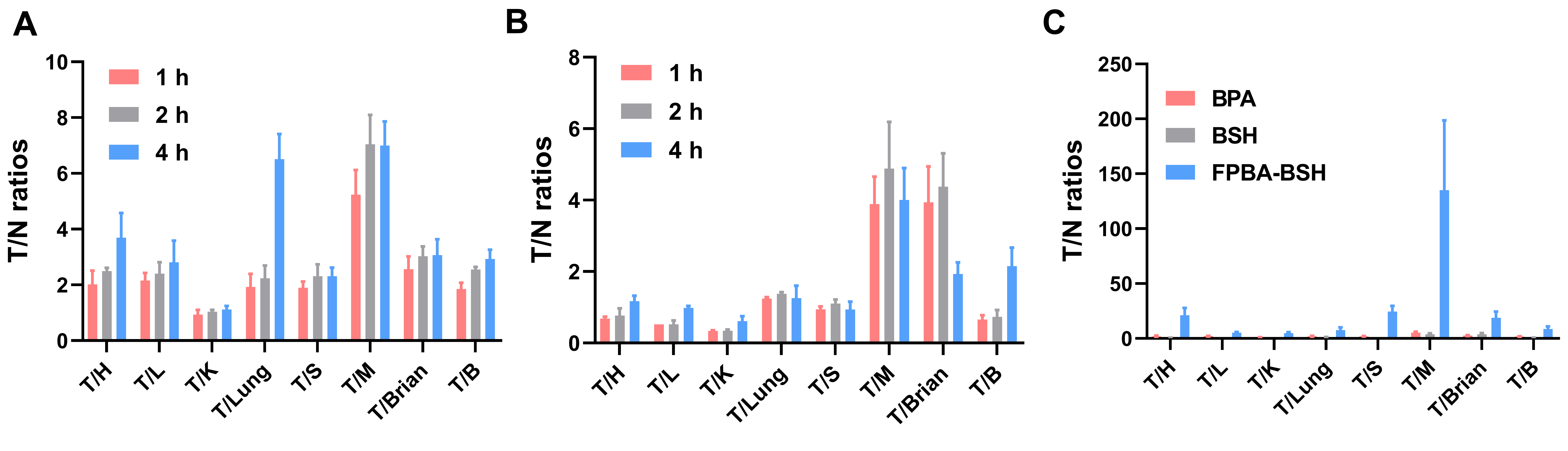


**Fig. S12** (A) T/N ratios for BPA at 1, 2, and 4 h post-administration. (B) T/N ratios for BSH at 1, 2, and 4 h. (C) T/N ratios for BPA, BSH, and FPBA-BSH at 1 h post-administration. (mean ± SD, *n* = 3).


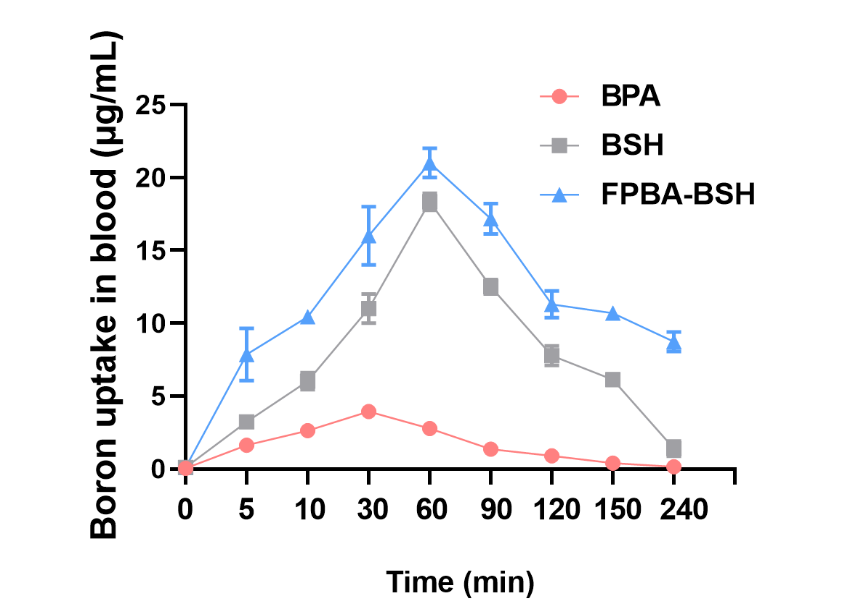


**Fig. S13** Blood pharmacokinetic profiles of BPA, BSH, and FPBA-BSH following systemic administration.

**Table S3.** Pharmacokinetic parameters of BPA, BSH, and FPBA-BSH in blood following systemic administration. The elimination half-life (t₁/₂) and systemic clearance (CL) were calculated from blood boron concentration–time profiles.

| Compound | T_1/2_ (min) | CL(L/(h.kg)) |
| --- | --- | --- |
| BPA | 58.2 | 0.42 |
| BSH | 115.5 | 0.13 |
| FPBA-BSH | 210.0 | 0.063 |


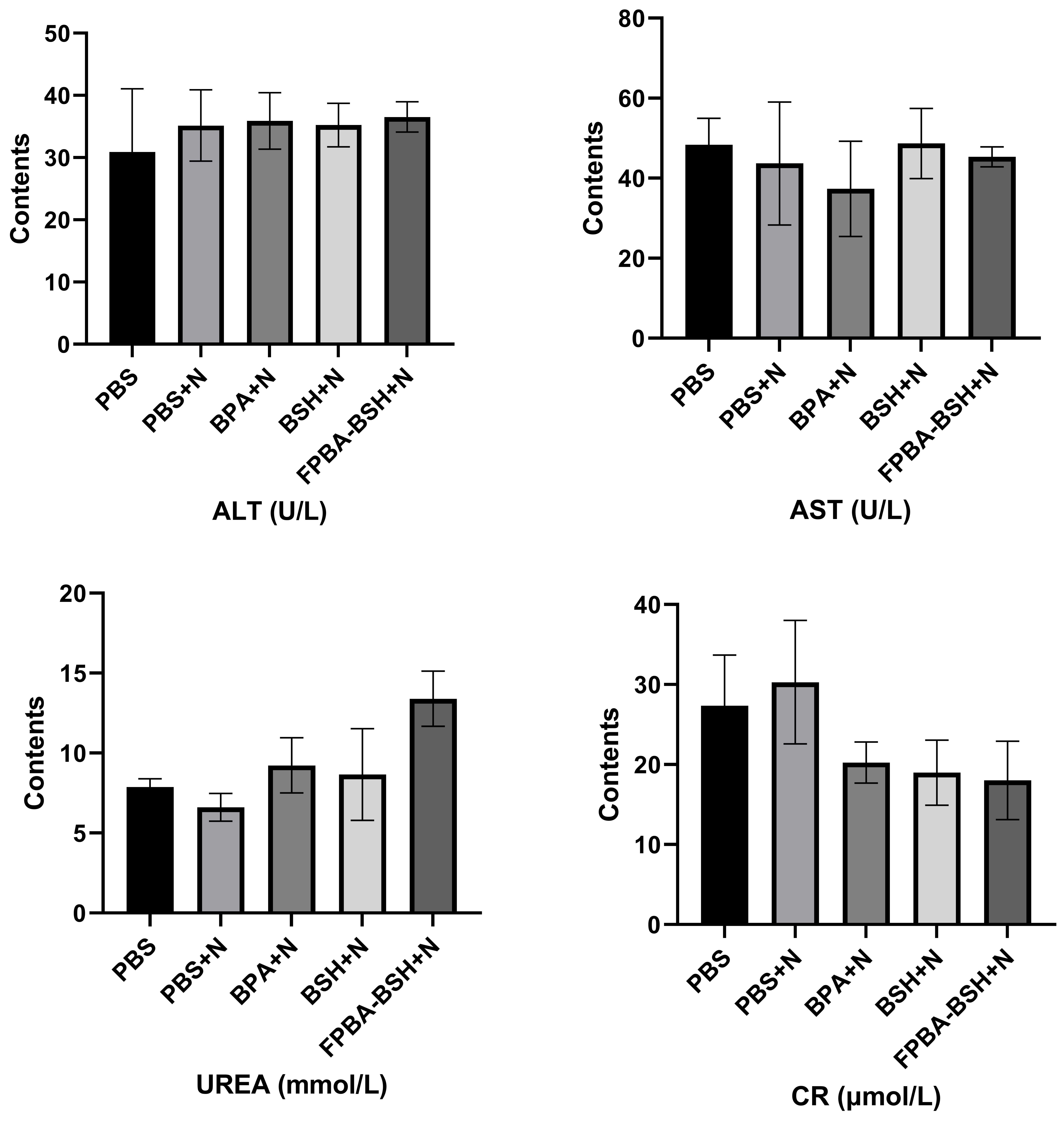


**Fig. S14** After the treatment, the blood biochemical indicators of the mice were tested. Data were presented as the mean value ± SD, *n* = 5.


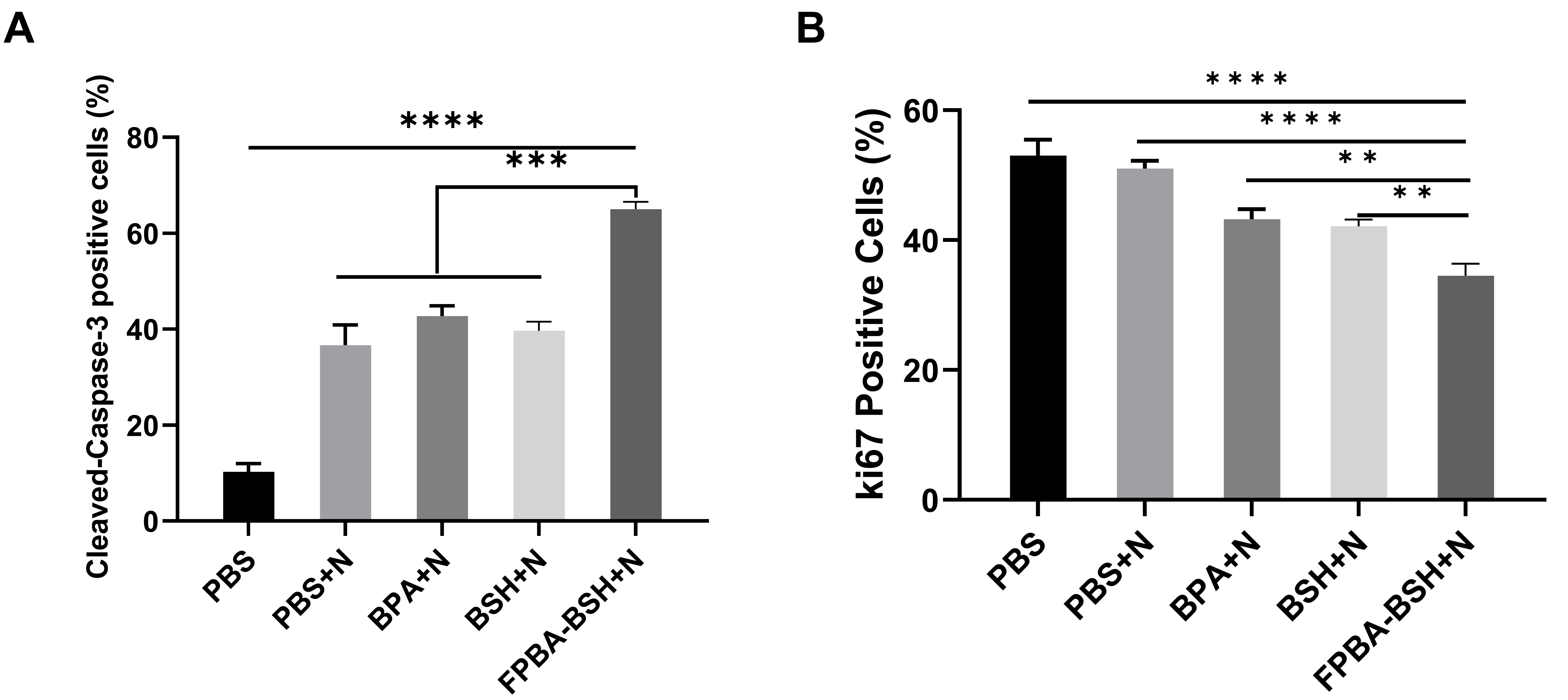


**Fig. S15** (A, B) Quantification of cleaved-Caspase-3 and Ki-67 expression levels. ***P* < 0.01, ****P* < 0.001, *****P* < 0.0001.


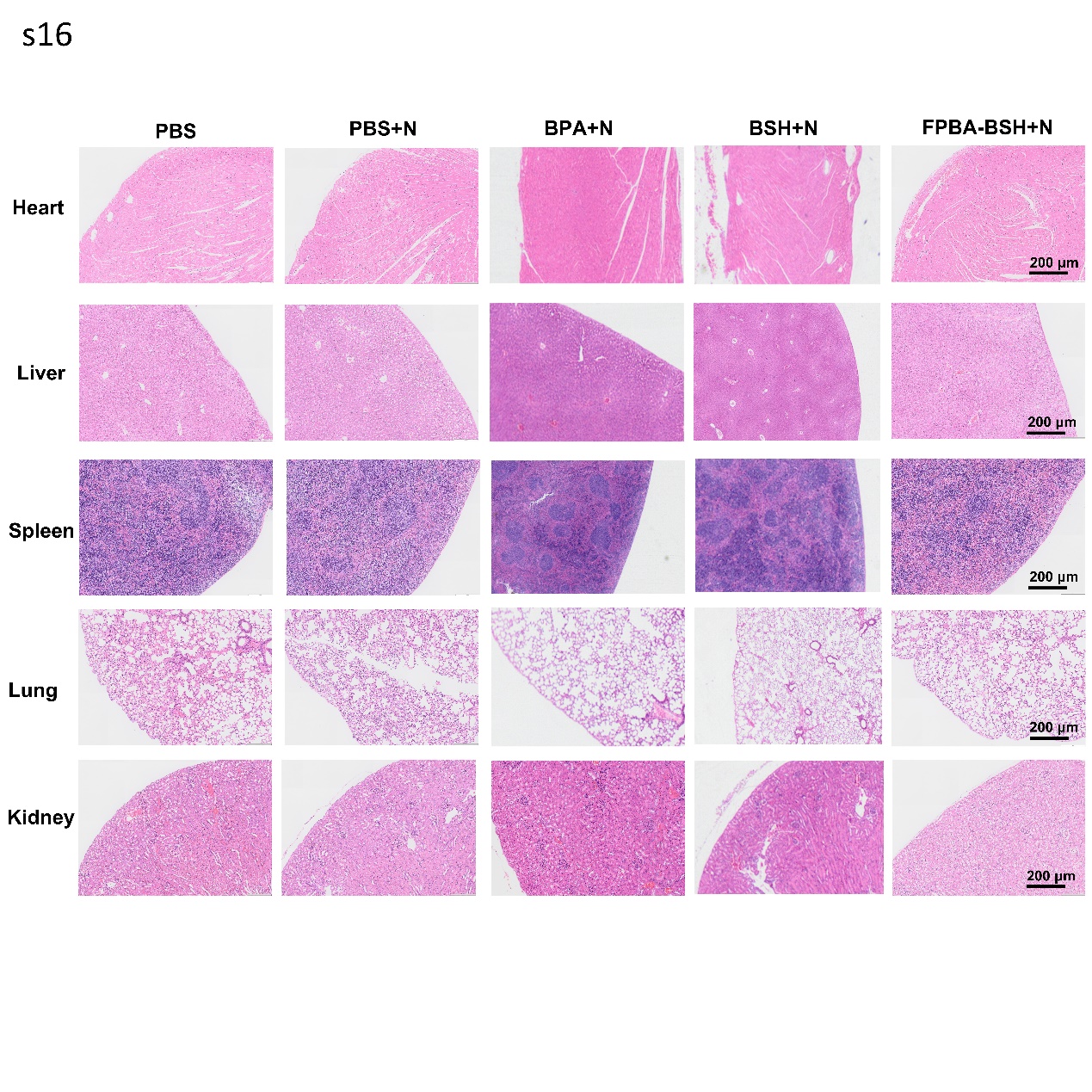


**Fig. S16** Safety evaluation of various treatments. The main organs (lungs, liver, brain, spleen, kidneys and heart) of the experimental mice collected after the treatment were stained with H&E. Scale bar = 200 μm.


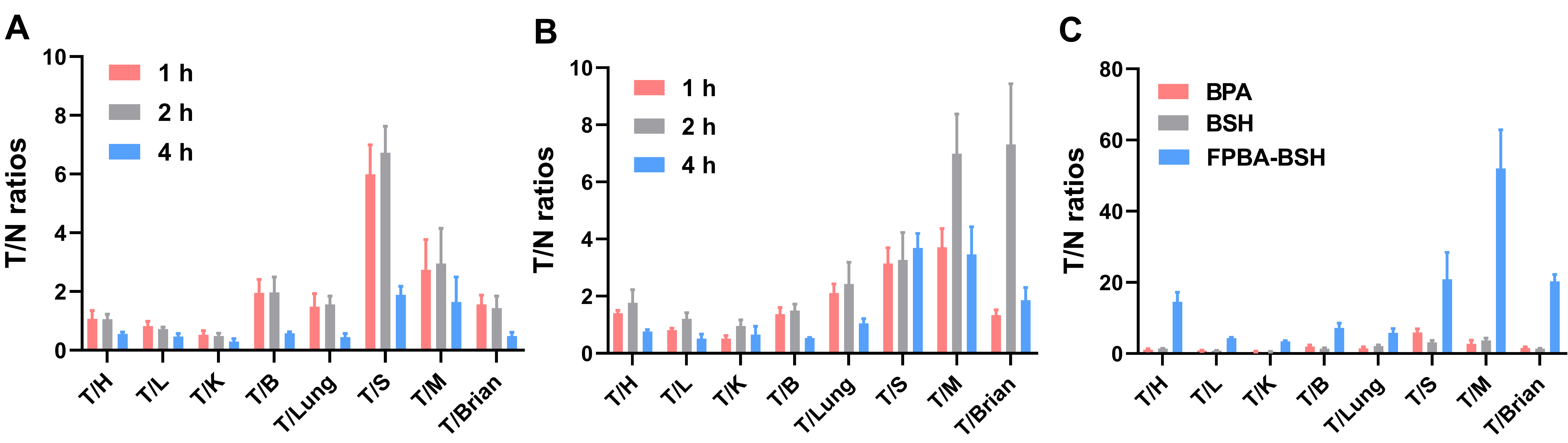


**Fig. S17** (A) T/N ratios for BPA at 1, 2, and 4 h post-administration. (B) T/N ratios for BSH at 1, 2, and 4 h. (C) T/N ratios for BPA, BSH, and FPBA-BSH at 1 h post-administration. (mean ± SD, *n* = 3).


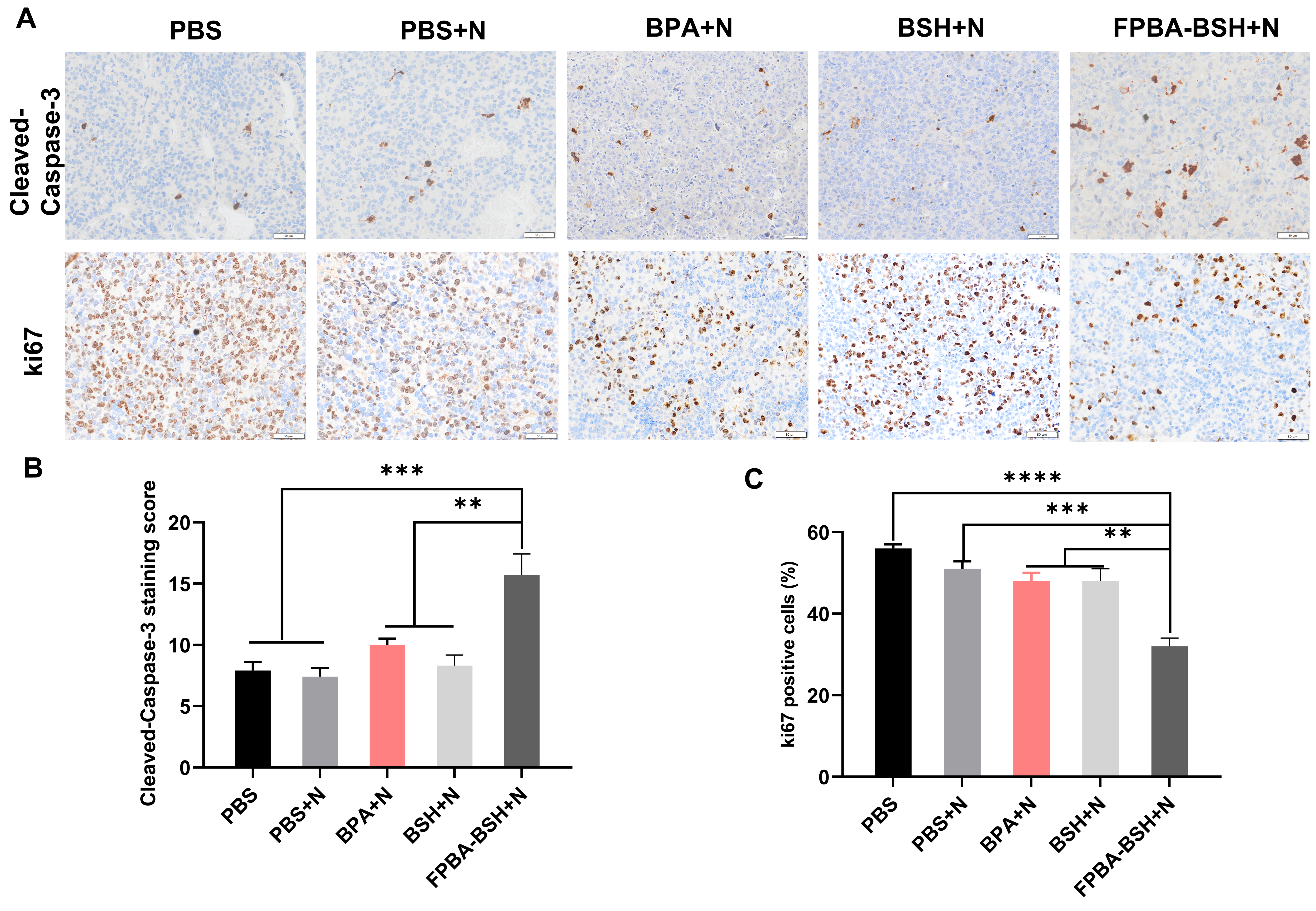


**Fig. S18** Evaluation of the therapeutic efficacy of FPBA-BSH in BNCT. (A) Cleaved- Caspase-3 in mouse brain tissue after BNCT. (B-C) Cleaved-Caspase-3, Ki-67 quantified statistical chart. Scale bar = 50 μm. ***P* < 0.01, ****P* < 0.001.


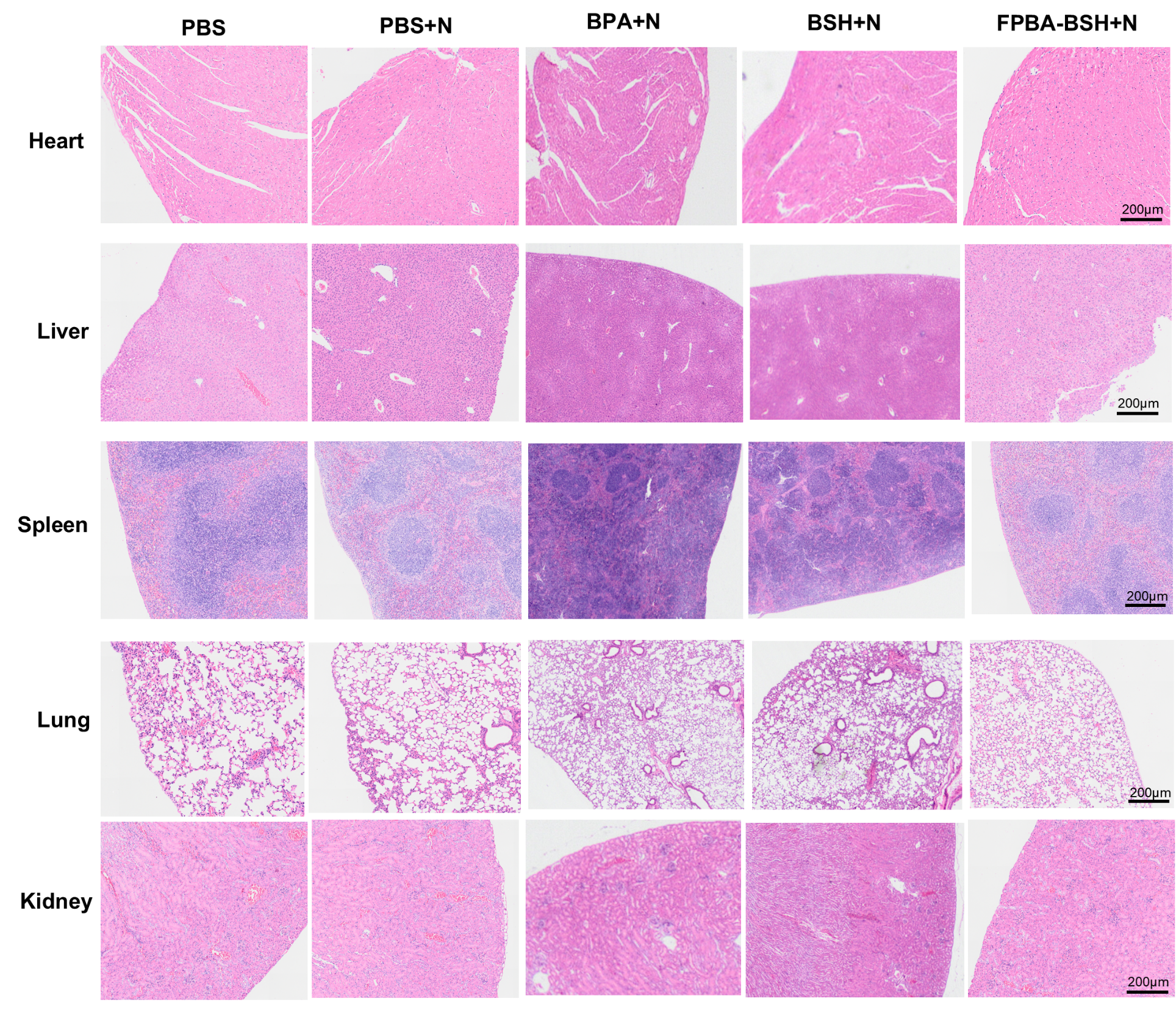


**Fig. S19** Safety evaluation of various treatments. The main organs (lungs, liver, brain, spleen, kidneys and heart) of the experimental mice collected after the treatment were stained with H&E. Scale bar = 200 μm.
